# Superconductivity of barium with highest transition temperatures in metallic materials at ambient pressure

**DOI:** 10.1038/s41598-023-50940-5

**Published:** 2024-01-16

**Authors:** Masaki Mito, Hiroki Tsuji, Takayuki Tajiri, Kazuma Nakamura, Yongpeng Tang, Zenji Horita

**Affiliations:** 1 Graduate School of Engineering, Kyushu Institute of Technology, Kitakyushu, 804-8550 Japan; 2 Faculty of Science, Fukuoka University, Fukuoka, 814-0180 Japan

**Keywords:** Superconducting properties and materials, Design, synthesis and processing

## Abstract

Pressure-induced superconductivity often occurs following structural transition under hydrostatic pressure (*P*_HP_) but disappears after the pressure is released. In the alkali-earth metal barium, superconductivity appears after structural transformation from body-centered cubic structure to hexagonal-close-packed (hcp) structure at *P*_HP_ = 5 GPa, and the superconducting transition temperature (*T*_c_) reaches a maximum of 5 K at *P*_HP_ = 18 GPa. Furthermore, by stabilizing the low-temperature phase at *P*_HP_ ~ 30 GPa, *T*c reached a higher level of 8 K. Herein, we demonstrate a significantly higher* T*_c_ superconductivity in Ba even at ambient pressure. This was made possible through severe plastic deformation of high-pressure torsion (HPT). In this HPT-processed Ba, we observed superconductivity at *T*_c_ = 3 K and *T*_c_ = 24 K in the quasi-stabilized hcp and orthorhombic structures, respectively. In particular, the latter* T*_c_ represents the highest value achieved at ambient pressure among single-element superconducting metals, including intermetallics. The phenomenon is attributed to a strained high-pressure phase, stabilized by residual strains generated from lattice defects such as dislocations and grain boundaries. Significantly, the observed *T*_c_ far exceeds predictions from DFT calculations under normal hydrostatic compressions. The study demonstrates the importance of utilizing high-pressure strained phases as quasi-stable superconducting states at ambient pressure.

## Introduction

Superconducting states, which are macroscopic quantum phenomena, are characterized by uniform electron wave function phases. Research into single elements is pivotal for new fundamental insights. Among the 118 elements in the periodic table, about one-fourth are ambient-pressure superconductors, with one-fifth becoming superconductors under hydrostatic pressure^1–4^. These are classified as *s*, *s*-*d*, *s*-*p*, and *s*-*f* electron systems, respectively. In alkali and alkali-earth metals, *s-*electrons significantly influence electric properties. First, Li and Be exhibit superconductivity at ambient pressure but at extremely low temperatures (*T*_c_ = 0.4 mK in Li^5^, *T*_c_ = 26 mK in Be^6,7^). In particular, for Li, the reduction in the structural symmetry in the unit cell at low temperatures is essential for superconductivity^2^. Heavier alkali and alkali-earth metals, such as Cs, Ca, Sr, and Ba, exhibit transition into *s*-*d* electron superconductors accompanying pressure-induced transformation from body-centered cubic (bcc) to other crystal structures^8–17^, where electron transfer from *s*- to *d*-orbitals plays a crucial role^2^. Extensive research, especially on Ba, focuses on hydrostatic contraction effects^9,17–20^. Thus, reduction in structural symmetry and structural phase transformation are crucial for stabilizing superconducting state in these metals. Therefore, manipulating lattice strain and crystal structures could feasibly enhance *T*_c_.

In this study, we utilized a high-pressure torsion (HPT) process to introduce shear and compressive strains. The graphical depiction of HPT, as displayed in Extended Data Fig. 1, illustrates that the magnitude of shear strain increases in proportion to the distance from the disk center, as well as the number of revolutions *N*. Herein, the compressive stress allows only the diagonal components in the strain tensor, whereas the shear strain leads to the nondiagonal components and plays an imperative role in generating possible crystal structures that realize a strain-induced superconducting state. In earlier studies, HPT processing of Re^21^ and Nb^22–24^ stabilized the superconductivity, and the increase in *T*_c_ for Re was due to the increase of the density of states (DOS) at the Fermi level with the expansion of the unit cell volume^21^, and that for Nb also to the DOS increase with the deformation of the unit cell^22–24^. In general, shear strain is known to introduce not only such simple structural deformations but also an accumulation of dislocations and leads to the formation of a fine-grained structure, resulting in a long-lived quasi-stable state even after releasing stress^25,26^.

In Ba, the presence of a variety of high-pressure phases under hydrostatic pressure (*P*_HP_)^16–20^ has been reported; in particular, a high-pressure phase Ba-VI (orthorhombic) stabilized at *P*_HP_ = 12–30 GPa and temperatures below 150 K appears to be the most suitable candidate for high *T*_c_^16,17^ (Extended Data Fig. 2). In this study, we utilize the HPT process to stabilize such high-pressure hcp and orthorhombic phases even under ambient pressure. Our findings demonstrate that these resulting high-pressure phases maintain their stability even after the stress is released. Notably, the structures achieved through HPT are significantly distinct from those derived sorely from normal hydrostatic compression. The strain at the unit-cell is a crucial factor for attaining superconductivity. Therefor, we anticipate that the HPT-processed Ba will generate appropriately stained structures, potentially leading to an unexpectedly high *T*_c_.

## Results

### Magnetic measurements and XRD analysis

**Figure 1:**
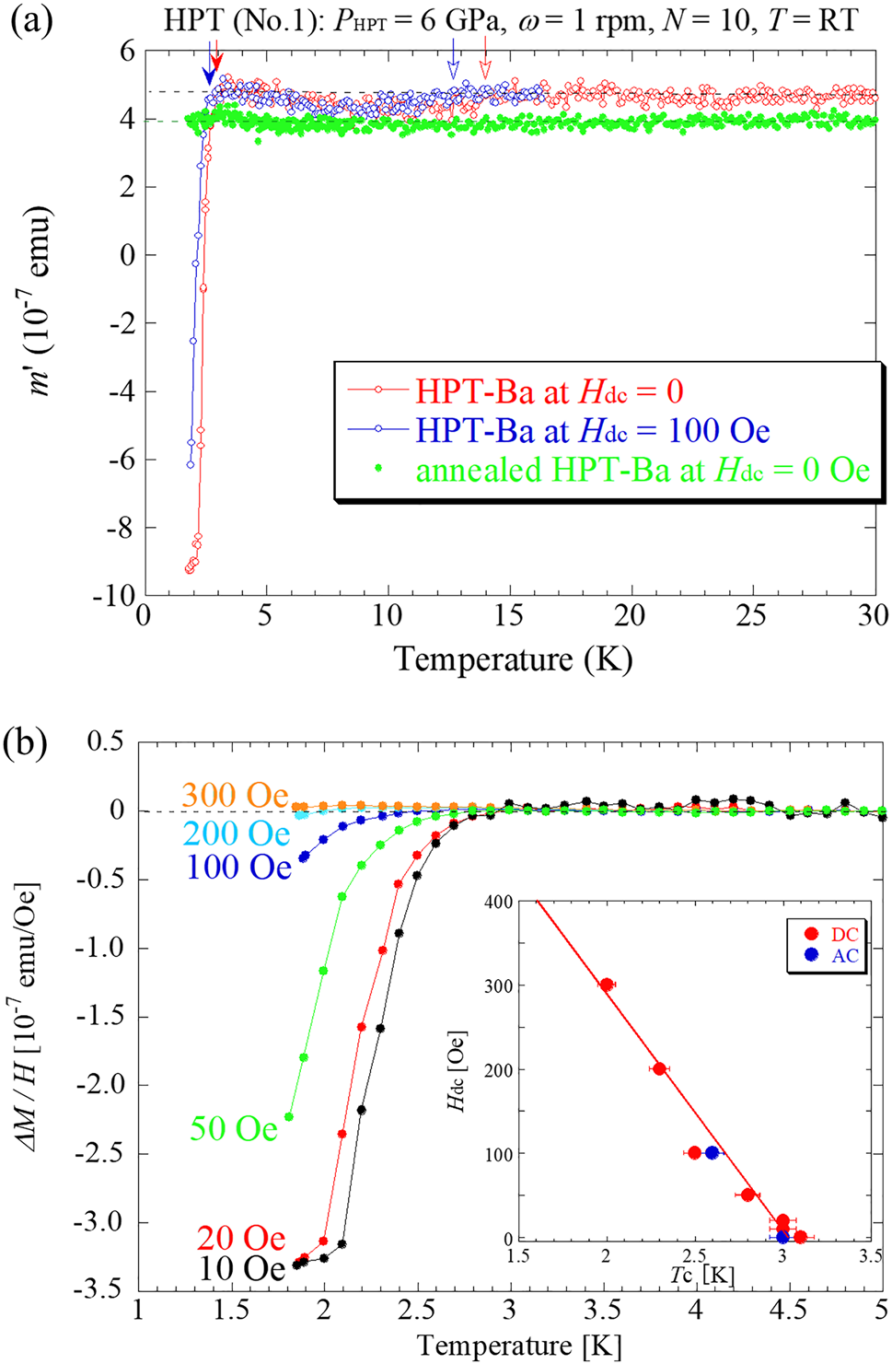
Temperature dependence of in-phase AC magnetization *m*’ (**a**) and DC magnetic susceptibility on *M*/*H* (**b**) for Ba subjected to HPT (No. 1) at room temperature (RT), where the pressure of HPT (*P*_HPT_) and revolution number (*N*) were 6 GPa and 10, respectively. During HPT, a Ba disk was encapsulated in an Al-based Al-3%Mg–0.2%Sc cell to prevent oxidization. (**a**) includes, for comparison, the results after annealing the HPT-processed Ba at 503 K in a vacuum for one hour. During the HPT processing at *P*_HPT_ = 6 GPa and RT, the targeted high-pressure phase was the hcp phase. The inset in (**b**) shows the dc magnetic field (*H*_dc_) dependence of *T*_c_ for *H*_d__c_ = 10-300 Oe.

Figure 1a shows the temperature (*T*) dependence of the in-phase AC magnetization *m*’ for HPT-processed Ba (*P*_HPT_ = 6 GPa, *N* = 10 at room temperature). *P*_HPT_ is the operational pressure used in HPT processing and differs from *P*_HP_, which is the usual hydrostatic pressure. A diamagnetic signal was unambiguously observed at 3 K in HPT-processed Ba. In addition, a small but visible diamagnetic signal was observed at 14 K. We confirmed that neither signal originated from the Al–Mg–Sc cells subjected to HPT processing. The diamagnetic signals shift to the low-temperature side when a DC magnetic field (*H*_dc_) of 100 Oe is applied. Figure 1a shows the results obtained after annealing the HPT-processed Ba at 503 K in a vacuum for one hour. No diamagnetic signals are observed in the sample after annealing. The annealing temperature of 503 K is more than half the melting point of Ba (999 K) and is sufficient to release the residual strain. Figure 1b shows the temperature dependence of the DC magnetization *M* for the same HPT-processed Ba. The onset temperature *T*_c_ for the lower-temperature diamagnetic anomaly exhibited a linear *H*_dc_ dependence, as shown in the inset of Fig. 1b. This phenomenon was also confirmed in the other samples (Extended Data Fig. 3). We note that the *T*_c_ of 3 K is approximately six times higher than that under the hydrostatic compression of *P*_HP_ = 6 GPa (Extended Data Fig. 2), thus indicating that the shear strain can induce an enhancement of *T*_c_ beyond normal compression. The volume fraction of the superconducting phase *V*_SC_ was evaluated to be at most 0.6%. Small amount of superconducting volume fraction originates from the large distribution of shear strain and the formation of small grains. The X-ray diffraction (XRD) pattern obtained by the reflection method, shown in Extended Data Fig. 4, reveals that multiple hcp phases existed with multiple bcc phases (see Extended Data Table 2). This feature was also confirmed in other HPT-processed Ba samples, although no diamagnetic signals were observed (sample No.3 in Extended Data Table 1), for which we think that, in the No. 3 sample, superconducting grains whose size was beyond the penetration length of the magnetic field would not occur. Commonly there is a variation of the lattice constant as 4.4–5.0 Å in the bcc structure, which is equivalent to the change in the atomic volume, *V*_atom_ = 63.4–41.8 Å^3^, corresponding to the range of *P*_HP_ = 0–6 GPa. In the residual hcp phases, *V*_atom_ comes to 38.1 and 27.6 Å^3^ which correspond to *P*_HP_ ~ 7 GPa (hcp #1) and ~ 18 GPa (hcp #2), respectively (see Extended Data Table 2).

**Figure 2:**
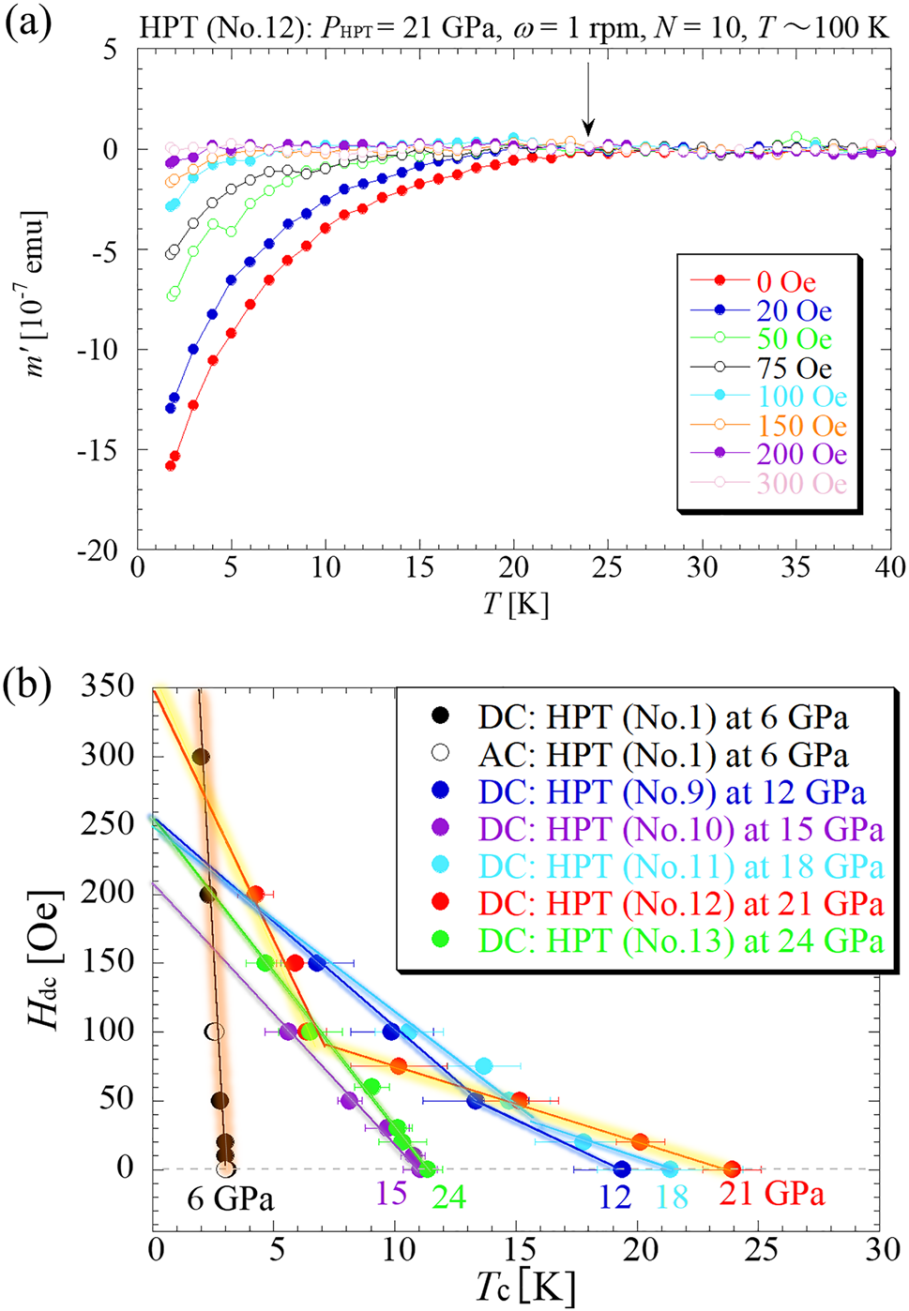
(**a**) Temperature dependence of the AC magnetization *m*’ for Ba subjected to HPT at liquid-nitrogen temperature, where the *P*_HPT_ value was 21 GPa. The targeted high-pressure phase is the orthorhombic phase. (**b**) *H*_dc_ dependence of *T*_c_ at *P*_HPT_ = 12, 15, 18, 21, and 24 GPa, along with that at *P*_HPT_ = 6 GPa (inset of Fig. 1b).

The superconducting properties of HPT-Ba were also investigated at cryogenic temperatures, where HPT processing was performed at *P*_HPT_ = 12–24 GPa for *N* = 10, and the processing temperature was maintained at approximately 100 K in liquid nitrogen. We found that HPT processing at cryogenic temperatures generated a high-pressure orthorhombic phase as a quasi-stable state (Extended Data No. 2). Figure 2a shows the *T* dependence of *m*’ for the HPT-processed sample at *P*_HPT_ = 21 GPa, where the magnetic shielding signal appears at approximately 24 K under *H*_dc_ = 0 Oe and disappears at *H*_dc_ = 300 Oe. The results for the other *P*_HPT_ are presented in Extended Data Fig. 5. Also, Fig. 2b compares the *H*_dc_ dependence of the onset *T*_c_ for the HPT samples processed under various *P*_HPT_ pressures, where we observe that the *P*_HPT_ = 21 GPa sample gives a highest *T*_c_ of 24 K. We emphasize that this *T*_c_ is the highest achieved at ambient pressure for pure metals, including intermetallics, although higher values of *T*_c_ than 24 K have been reported under the application of high pressures such as scandium (36 K at 260 GPa)^27^, titanium (26 K at 240 GPa)^28,29^, and calcium (29 K at 220 GPa)^30^. The *T* dependence of *m*’ in Fig. 2a is rather broad compared to that in Fig. 1a. This is because of the difference in the operating temperature during HPT processing. In general, lowering the operating temperature during HPT processing can result in the loss of sample viscoelasticity, and the HPT processing of such samples produces small sizes and diverse phases. Conversely, HPT processing under high-temperature conditions generates samples with large domains.

XRD analysis, conducted through the reflection method, was performed on HPT-processed samples at cryogenic temperatures under *P*_HPT_ = 12, 21, and 24 GPa. We found the coexistence of several orthorhombic, hcp, and bcc phases, as shown in Extended Data Figs. 6–8. The coexistence of these phases was also confirmed using thrubeam XRD, as shown in Extended Data Figs. 9–13. The results of the XRD analysis are summarized in Extended Data Tables 2–3. In HPT processing, many structures with the same crystal symmetry were generated, and different lattice parameters reflected the spatial distribution of the shear strain, as mentioned in detail in the Discussion and Methods sections. Anyway, metastabilization of the high-pressure phase via the residual strain is one of the striking character of the HPT processing. Also, the volume fractions of the high-pressure phases observed in the reflective XRD experiments (Extended Data Table 2) were higher than those detected through the thrubeam method (Extended Data Table 3). This discrepancy suggests that high-pressure phases are more likely to form near the material’s surface, highlighting a unique aspect of material manipulation via HPT processing. Additionally, the magnetic shielding signal was relatively small in the nanosized grain materials, primarily because the magnetic flux can be penetrated near the surface, thus making the Meissner signal to be weakened, even if the grain has superconducting properties. Therefore, the actual *V*_SC_ can be evaluated to be more than the value via the magnetic measurements.

### Phase diagram

**Figure 3:**
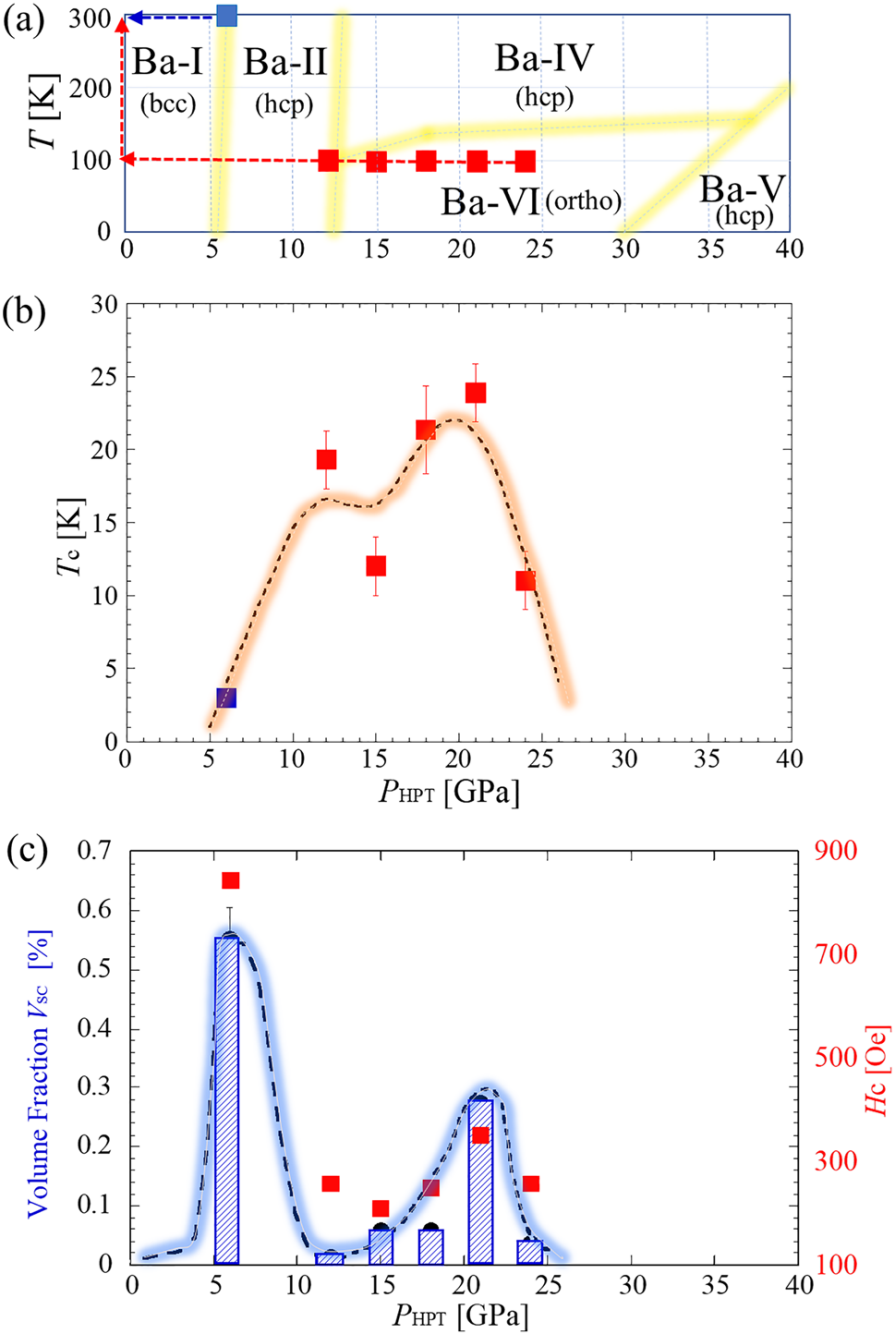
(**a**) Schematic *P*-*T* phase diagram of Ba, where the blue and red squares specify the temperature and operating pressure *P*_HPT_ during the HPT processing. After the HPT processing, the samples are returned to the room temperature and ambient pressure, which is represented as the arrows in (**a**). *P*_HPT_ dependence of *T*_c_ (**b**), and volume fraction *V*_SC_ and *H*_c_ (**c**). Notice that the horizontal axis in the panels (**b**) and (**c**) is not the hydrostatic pressure *P*_HP_, but the operating pressure *P*_HPT_ during the HPT processing.

Figure 3 summarizes the obtained results. As illustrated in Fig. 3a, a thermodynamic *P*_HP_-*T* phase diagram of Ba is presented, which includes the sequence of *P*_HPT_ after the revolution processing. Figure 3b, c show the obtained *T*_c_ and *V*_SC_ as a function of *P*_HPT_, respectively. It is important to note that the horizontal axis in Fig. 3b, c is *P*_HPT_ not *P*_HP_, and the *T*_c_ measurements were performed at ambient pressure. From panel (b), we observe *T*_c_ values exceeding 10 K for *P*_HPT_ values greater than 12 GPa, and *T*_c_ = 20–25 K at approximately 10 < *P*_HPT_ < 20 GPa. In addition, from panel (c), the largest *V*_SC_ occurs at *P*_HPT_ = 6 GPa, which is consistent with the trend that HPT processing at high temperatures can generate larger grains compared to those at lower temperatures^31–34^, as mentioned above. We infer that the actual *V*_SC_ for the sample processed using liquid-N_2_ temperature HPT at *P*_HPT_ = 21 GPa would approach to that of the sample processed using room-temperature HPT at *P*_HPT_ = 6 GPa.

Proving superconductivity through electrical resistance measurement is challenging, as *V*_SC_ is at most 1%. Additionally, the surface of Ba tends to oxidize slightly during HPT processing and preparation. Preparing electrodes on a room-temperature surface may release residual strain. Therefore, contactless magnetic measurements are more suitable for confirming superconductivity.

### Ab initio calculation

**Figure 4:**
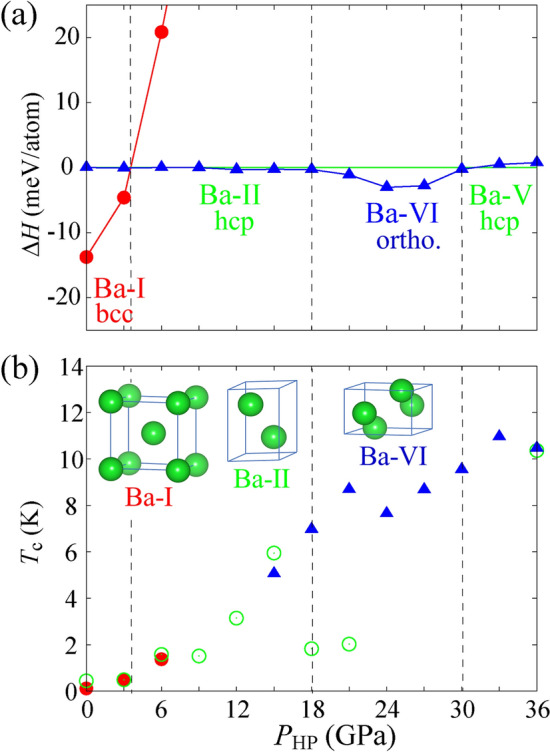
(**a**) Structural stability of the bcc (red circles), hcp (green line), and orthorhombic (blue triangles) phases as a function of the hydrostatic pressure *P*_HP_, where we plot the enthalpy relative to the hcp phase. (**b**) *P*_HP_ dependence of our calculated *T*_c_, where *T*_c_ was calculated for the most stable phase at a given hydrostatic pressure.

To understand the basic properties of Ba, ab initio density functional calculations were performed with normal hydrostatic compressions. Figure 4a shows the enthalpies of the bcc and orthorhombic phases relative to the hcp phase as a function of *P*_HP_. The orthorhombic unit cell is equivalent to a double-hcp unit cell. For *P*_HP_ < 4 GPa, the bcc phase (red dots) is stable; however, as the pressure increases to *P*_HP_ > 4 GPa, the hcp phase becomes stable. At 18 < *P*_HP_ < 31 GPa, the orthorhombic phase (blue triangles) becomes stable, whereas at *P*_HP_ > 31 GPa, the hcp phase becomes stable again. This trend is the same as that reported in previous density functional calculations^16^. Figure 4b shows the pressure dependence of the calculated *T*_c_ based on the McMillan–Allen–Dynes formula^35,36^. *T*_c_ gradually increases with pressure and reaches 10–11 K at approximately *P*_HP_ = 30 GPa, which is nearly consistent with the experimental results for *T*_c_ = 8 K at *P*_HP_ = 30 GPa compressed at 10 K^17^. It is known from ab initio calculations that *T*_c_ decreases monotonically at pressures higher than *P*_HP_ = 50 GPa^37^. Thus, the *T*_c_ maximum under normal hydrostatic compression is only of the order of 10 K, which is clearly smaller than the present *T*_c_ maximum (approximately 20–25 K) attained in HPT-processed Ba. The reason for this *T*_c_ difference remains unclear, and leaves a nontrivial and important issue. As long as we suppose Ba to be a phonon-based superconductor, we must consider that HPT processing works to strengthen the effective electron-lattice interactions.

## Discussion

The structural optimization of ab initio density functional calculations provides the structural parameters as follows: bcc (Ba-I) with *a* = 5.0263(1) Å at *P*_HP_ = 0 GPa, hcp (Ba-II) with *a* = 3.6394 (5) Å and *c* = 5.3921(1) Å at *P*_HP_ = 15 GPa, and orthorhombic (Ba-VI) *a* = 5.8269(7) Å, *b* = 5.0866 (7) Å, and *c* = 3.3639(2) Å at *P*_HP_ = 30 GPa. These lattice parameters are consistent with the experimental values under the corresponding *P*_HP_^16,38^. First, for the surface region of HPT-Ba under *P*_HPT_ = 6 GPa at room temperature, it consists mainly of multiple bcc structures with *V*_atom_ corresponding to ≤ 6 GPa as seen in Extended Data Table 2: In bcc #1, strain is perfectly released, and in bcc #4, the strain corresponding to *V*_atom_ at *P*_HP_ = 6 GPa remains. *P*_HP_’s for *V*_atom_ of bcc #2 and #3 are approximately *P*_HP_ = 2 and 5 GPa, respectively. Furthermore, the hcp phases remain, which correspond to the crystal lattices at *P*_HP_ = 7 (hcp #1) and 18 GPa (hcp #2). As shown in Fig. 4, the *T*_c_ = 3 K superconductivity of HPT-Ba at *P*_HPT_ = 6 GPa is attributed to hcp #1 and #2. The inner region does not include bcc #4 or hcp #2, as shown in Extended Data Table 3.

Next, for the cryogenic temperature HPT-Ba, the sample surface is composed of ortho #2, corresponding to *P*_HP_ > 20 GPa, and ortho #1 and ortho’ #1, corresponding to *P*_HP_ =  + 3 to −1 GPa, respectively. The inner region also exhibits a similar composition; however, an additional bcc #6 corresponding to *P*_HP_ = 13 GPa exists, and approximately half of the specimen volume recovers bcc #1. The details of the residual phases for *P*_HPT_ = 21 GPa are shown in Extended Data Fig. 14a. Referring to Fig. 4 and Extended Data Fig. 14a, the *T*_c_ > 10 K superconductivity is attributed to strained ortho #2; however, to explain *T*_c_ ~ 20 K, an additional factor is needed.

## Conclusion

In summary, we successfully stabilized high-pressure Ba hcp and orthorhombic phases at ambient temperature and pressure using HPT processing. The high-pressure phases, hcp and orthorhombic, are more conductive to superconductivity compared to the original bcc phase. Hence, phase stabilization via HPT processing effectively endows Ba with new superconducting properties at ambient pressure. Though preset in small fractions, superconducting transitions as high as 24 K were observed in HPT-processed Ba at ambient pressure. Initially, HPT processing at room temperature led to the discovery of the Ba-II hcp phase with a *T*_c_ of ~ 3 K. Subsequent HPT processing at cryogenic temperatures using liquid nitrogen resulted in an increased volume fraction of the strained Ba-VI orthorhombic phase with a *T*_c_ of ~ 24 K, the highest *T*_c_ ever observed for single-element superconductors at the ambient pressure, except for pressured system. The relationship between the stain nature in the strained phase and the high *T*_c_ must be investigated more carefully and microscopically. Contrary to the traditional belief that shear strain negatively impacts the stabilization of the superconducting state by reducing grain size and crystallinity, our study proposes a novel method for developing high *T*_c_ superconductors, opening up significant opportunities for advancing electronic property-related functionalities. Ab initio calculations had suggested a *T*_c_ of approximately 10 K under normal hydrostatic compression, aligning with previous experimental studies. However, structural analysis of the HPT-processed samples revealed a coexistence of multiple strained phases, and the understanding the electronic states of these strained quasi-stable states is crucial for investigating the origin of the high *T*_c_ observed in HPT-processed Ba.

## Methods

### High pressure torsion

Processing through high-pressure torsion (HPT) causes severe plastic deformation, as shown in Extended Data Fig. 1^31–34^. First, a disk sample was uniaxially compressed between opposite-faced anvils, which were made of WC (SR16C, NOTOALLOY Co., Ltd.), using a 50-ton press machine. The lower-side anvil was then rotated with respect to each other for *N* revolutions. During the revolution process, severe shear strain accumulates, and its magnitude increases in proportion to the distance from the disk center and the number of revolutions. In the early stage, the grain size was reduced, and subsequent recrystallization proceeded, resulting in a balance between these effects.

In this study, HPT was first conducted at room temperature for *N* = 10 turns under a pressure of *P*_HPT_ = 6 GPa with a rotation speed of 1 rpm. In this room-temperature HPT process, Ba was easily oxidized and sealed in an Al-based Al-3%Mg-0.2%Sc (Al–Mg–Sc) alloy cell under an Ar atmosphere. A Ba disk (purity 99.9%, 7-mm-diameter, 0.6-mm-thickness) in the Al-alloy cell was then processed using an HPT machine.

The HPT process was also conducted using disks with dimensions of 5 mm in diameter and 1 mm in thickness so that it was possible to control the applied pressure up to a maximum of *P*_HPT_ = 24 GPa. The HPT process was performed in liquid nitrogen, where liquid and gaseous nitrogen played a role in preventing the oxidation of the Ba disks instead of the Al-alloy cell. A thermocouple was inserted into the upper anvil at a position close to the sample^39^ such that the processing temperature was maintained at approximately 100 K during the HPT operation in liquid nitrogen^40^. The thickness after the HPT processing was reduced to 0.54 mm. We previously reported that the grain size after HPT processing under *P*_HPT_ at 20 GPa was approximately 100 nm for pure Re^21^. In this experiment, the corresponding HPT processing was conducted at 100 K, resulting in the accumulation of intense strain and the formation of ultrafine grains whose size is much smaller than 100 nm.

Thus, HPT processing was conducted for the state represented by the closed boxes in Fig. 3a. After HPT, the samples were removed from the HPT apparatus via the route represented by the arrows and maintained at 77 K in a liquid nitrogen vessel. The samples were removed from the vessel immediately before the magnetic measurements and XRD experiments. For the magnetic measurements, the samples were placed under ambient pressure conditions, and the temperature was cooled to 1.8 K. XRD experiments were conducted under ambient pressure and room temperature on samples coated with paraffin oil to prevent oxidization.

After the HPT processing, a disk specimen with 4 mm diameter and 0.7 mm thickness and a fragment specimen with a volume < 0.1 × 0.1 × 0.1 mm^3^ were prepared from the outer-side part excluding the center part for magnetic measurements. The mass of Ba was estimated correctly, where the volume of Ba (maybe 0.3-mm-thickness) could be roughly evaluated. Thirteen specimens were prepared using HPT at both room temperature and approximately 100 K. A summary of these specimens is presented in Extended Table 1.

### Magnetic measurements

The *T* dependence of the in-phase AC magnetization (*m*') was observed using a commercial superconducting quantum interference device (SQUID) magnetometer (Quantum Design Co. Ltd.) at an AC magnetic field of 3.86 Oe and 10 Hz under certain DC magnetic fields (*H*_dc_). The AC magnetization is intrinsically different from the AC magnetic susceptibility via electromagnetic induction. The former detects the magnetization itself following the AC field, whereas the latter does the time derivative of the magnetization under the AC field.

Temperature dependence of the DC magnetization (*M*) was also observed at some *H*_dc_'s. It should be noted that the HPT-processed samples contained some residual strain even after releasing the stress subjected by HPT processing, whereas their magnetic measurements were performed under ambient pressure conditions. The shear strain increases proportionally to the radial distance from the center of the HPT-processed disks^31–34^, such that the broadening of the magnetic shielding signal originates from the wide distribution of the intensity of the residual strain.

### X-ray diffraction

We performed reflection-type X-ray diffraction (XRD) at room temperature using SmartLab (Rigaku) para-focusing optics with Cu (Kα_1_ and Kα_2_) radiation. We also used thrubeam-type XRD analysis using synchrotron radiation with an X-ray energy of 60 keV at the beamline (BL04B1) of SPring-8 at the Japan Synchrotron Radiation Research Institute (JASRI), and 16 keV at the beamline (BL-8B) of the Photon Factory at the Institute of Materials Structure Science, High Energy Accelerator Research Organization. The observed diffraction peaks were deconvolved using the Voight function into those associated with each Ba phase by multipeak fitting. The lattice constants were calculated from the relationship between the lattice constants and the plane indices determined from the Bragg peak angles of the deconvoluted peaks. All the crystal structures were evaluated based on the assumption that the atomic positions did not change.

The reflection type mainly detects crystal structures at the surface area, whereas the thrubeam type detects the entire area across the thickness, including the surface. The XRD patterns of the samples subjected to HPT processing at cryogenic temperatures are shown in Extended Data Fig. 6–13, and the analytical results are presented in Extended Data Tables 2–3. The hydrostatic pressure *P*_HP_ corresponding to the atomic volume *V*_atom_ was estimated using Extended Data Fig. 14, which was constructed from the XRD data reported in the literature at a hydrostatic pressure^38^.

Herein, we describe the crystal structure of HPT-processed Ba obtained using these two methods. First, we picked up important experimental facts in the reflection method (see Extended Data Table 2) for the Ba specimens subjected to room-temperature HPT at *P*_HPT_ = 6 GPa, whose conditions are suitable for stabilizing the hcp phase called the Ba-II phase: (1) More than half of the sample volume is a bcc phase, and they have an atomic volume *V*_atom_ corresponding to *P*_HP_ < *P*_HPT_. The smallest *V*_atom_ corresponds to* P*_HP_ = 5.3 GPa. The maximum *P*_HP_ = 5.3 GPa did not exceed *P*_HPT_ 6 GPa. (2) Most of the bcc phases were bcc#1, corresponding to *P*_HP_ = 0 GPa. (3) The hcp phase also existed with *V*_atom_ corresponding to *P*_HP_ > *P*_HPT_ = 6 GPa (i.e., 7 and 18 GPa), and their volume fractions were almost equal. Next, in the thrubeam method (see Extended Data Table 3), (1) More than two-thirds are the bcc phases, which have *V*_atom_ corresponding to *P*_HP_ ≤ 2.5 GPa. (2) A single hcp phase with *V*_atom_ corresponding to *P*_HP_ = 7 GPa was observed. Summarizing the difference between the two methods, the surface regions had more varied and compressed phases than those in the interior.

In HPT-processed Ba at cryogenic temperatures, the *P*_HPT_ values varied from 12 to 24 GPa. These conditions are suitable for stabilizing the orthorhombic phase, which is called the Ba-VI phase. At first, we pick up important experimental facts in the reflection method (see Extended Data Table 2): (1) Approximately half of the sample volume belongs to bcc phases with *V*_atom_ corresponding to *P*_HP_ ≤ 5.0 GPa. This feature was commonly observed in room-temperature HPT-Ba at *P*_HPT_ = 6 GPa. The value of *P*_HPT_ = 5 GPa is consistent with the upper threshold value of the *P*_HP_ region for the Ba-I phase. This suggests that approximately half of the compressed Ba sample recovered the bcc state of the Ba-I phase during the stress release after HPT processing. The residual bcc states were not uniform, and some candidates corresponded to *P*_HP_ = 0–5 GPa. (2) The hcp phase remained in the stress-release process after HPT processing. The hcp phase has *V*_atom_ corresponding to approximately *P*_HP_ = 12 GPa, which is close to the threshold value of *P*_HP_ between Ba-II (hcp) and Ba-VI (ortho). This phenomenon was commonly observed in samples with *P*_HPT_ = 12, 21, and 24 GPa. (3) The two types of orthorhombic structures were stabilized after HPT processing. One is ortho #1 and #2, with the space group *P*nma, which is the same as the structure of Ba-VI stabilized under hydrostatic pressure^16,17^. The other is ortho’ #1, with the space group of *C*mcm, created by shear strain under severe compression at cryogenic temperatures. Thus, three states exist: ortho #1, ortho #2, and ortho’ # 1. Both ortho #1 and ortho’ #1 have *V*_atom_ corresponding to *P*_HP_ ~ 0 GPa, and they cannot be created by typical hydrostatic compression. This is simply the effect of the shear stress under severe compression at cryogenic temperatures. In contrast, ortho #2 has *V*_atom_ corresponding to *P*_HP_ = 22–26 GPa. Next, in the thrubeam method (see Extended Data Table 3), new phenomena appear compared to those observed in the reflection method: (1) new bcc states with *V*_atom_ corresponding to *P*_HP_ ~ 13 GPa appear, and the volume fraction occupies approximately 10%. (2) At *P*_HPT_ = 12, 15, and 18 GPa, the hcp phase only has a state with *V*_atom_ corresponding to *P*_HP_ ~ 12 GPa, which is close to the threshold value of *P*_HP_ between Ba-II (hcp) and Ba-VI (ortho). However, for larger *P*_HPT_ conditions with *P*_HPT_ = 21 and 24 GPa, there also appears the hcp state with *V*_atom_ corresponding to *P*_HP_ = 14–16 GPa. (3) Three orthorhombic states were confirmed in the interior, and ortho #2 has *V*_atom_ corresponding to *P*_HP_ = 19–24 GPa. The volume fraction of the ortho #2 phase in the interior is smaller than that on the surface.

Given the above experimental facts, we can imagine the appearance of quasi-stable states during the stress-release process after HPT.

### Ab initio calculations

To analyze the structural stability of the bcc, hcp, and orthorhombic phases of Ba at a given external pressure, we performed ab initio density functional calculations for these systems using the QUANTUM ESPRESSO package^41,42^. We used the Perdew–Burke–Ernzerhof type^43^ for the exchange–correlation functional, and norm-conserving pseudopotentials were generated by the code ONCVPSP (Optimized Norm-Conserving Vanderbilt PSeudopotential)^44^ and obtained from PseudoDojo^45^. We used a 20 × 20 × 20 Monkhorst–Pack k-mesh for the Brillouin zone integration. The kinetic energy cutoff was set to 144 Ry for the wave functions and 576 Ry for the charge density. The Fermi energy was estimated by Gaussian smearing with a width of 0.136 eV^46^. The crystal structures were optimized with a total energy convergence better than 1 meV/atom. We verified that these conditions ensured a better convergence and enabled a quantitative comparison of the enthalpies of each phase. The phonon modes were calculated using the density-functional linear-response method^47,48^. The calculation conditions for the electron–phonon coupling are as follows: For the bcc, 14 × 14 × 14 k-mesh and 7 × 7 × 7 q-mesh are used for the electronic structure and phonon mode calculations, respectively. To ensure the convergence of the electron–phonon-coupling matrix elements, a large 28 × 28 × 28 k-mesh (k_fit_) was used with Gaussians of width 0.136 eV. For hcp, the sizes of the k-mesh, q-mesh, and k_fit_-mesh were 12 × 12 × 12, 6 × 6 × 6, and 24 × 24 × 24, respectively. For the orthorhombic phase, the k-mesh, q-mesh, and k_fit_-mesh values were 12 × 12 × 12, 4 × 4 × 4, and 24 × 24 × 24, respectively. The superconducting transition temperatures were estimated using the McMillan–Allen–Dynes formula^35,36^ with an effective Coulomb pseudopotential (μ* = 0.1). Electron–phonon coupling λ and characteristic frequency ω_ln_ were numerically evaluated using the frequency integral of the Eliashberg function. Density functional calculations prove that an orthorhombic structure based on *P*nma can be ideal for realizing a high *T*_c_ superconducting state in Ba.

### Supplementary Information

Supplementary Information.

## Data Availability

Data supporting the findings of this study are available from the corresponding authors upon reasonable request.

## References

[1] Ashcroft NW (2002). Putting the squeeze on lithium. Nature.

[2] Schilling, J. S. High-Pressure Effects, in Handbook of High-Temperature Superconductivity, edited by J: R: Schrieffer, pp.427–462 (Springer, New York, 2007).

[3] Hamlin, J. J. Superconductivity in the metallic elements at high pressures, *Physica C* (Amsterdam, Neth.) **514** (2015) 59–76.

[4] Debessai M, Hamlin JJ, Schilling JS (2008). Comparison of the pressure dependences of *Tc* in the trivalent *d*-electron superconductors Sc, Y, La, and Lu up to megabar pressures. Phys. Rev. B.

[5] Tuoriniemi, J., Juntunen-Nurmilaukas, K., Uusvuori, J., Pentti, E., Salmela, A., & Sebedash, A. Superconductivity in lithium below 0.4 millikelvin at ambient pressure, *Nature* **447** (2007) 187–189. 10.1038/nature05820 17495921

[6] Falge RL (1967). Superconductivity of hexagonal beryllium. Phys. Lett. A.

[7] Soulen RJ, Colwell JH, Fogle WE (2001). The superconductive critical magnetic field of beryllium. J. Low. Temp. Phys..

[8] Wittig J (1970). Pressure-induced superconductivity in cesium and yttrium. Phys. Rev. Lett..

[9] Shimizu K, Amaya K, Suzuki N (2005). Pressure-induced superconductivity in elemental materials. J. Phys. Soc. Jpn..

[10] Olijnyk H, Holzapfel WB (1985). High-pressure structural phase transition in Mg. Phys. Rev. B.

[11] Struzhkin VV, Eremets MI, Gan W, Mao HK, Hemley RJ (2002). Superconductivity in dense lithium. Science.

[12] Sin'ko GV, Smirnov NA (2005). Relative stability and elastic properties of hcp, bcc, and fcc beryllium under pressure. Phys. Rev. B.

[13] Nakamoto Y, Yabuuchi T, Matsuoka T, Shimizu K, Takemura K (2007). Crystal structure and electrical property of calcium under very high pressure. J. Phys. Soc. Jpn. Suppl. A.

[14] Ma Y, Eremets M, Oganov AR, Xie Y, Trojan I, Medvedev S, Lyakhov AO, Valle M, Prakapen-ka V (2009). Transparent dense sodium. Nature.

[15] Kim DY, Srepusharawoot P, Pickard CJ, Needs RJ, Bovornratanaraks T, Ahuja R, Pinsook U (2012). Phase stability and superconductivity of strontium under pressure. Appl. Phys. Lett..

[16] Desgreniers S, Tse JS, Matsuoka T, Ohishi Y, Li Q, Ma Y (2015). High pressure–low temperature phase diagram of barium: Simplicity versus complexity. Appl. Phys. Lett..

[17] Jackson DE, VanGennep D, Vohra YK, Weir ST, Hamlin JJ (2017). Superconductivity of barium-VI synthesized via compression at low temperatures. Phys. Rev. B.

[18] Wittig J, Matthias BT (1969). Superconductivity of barium under pressure. Phys. Rev. Lett..

[19] Moodenbaugh AR, Wittig J (1973). Superconductivity in the high-pressure phases of barium. J. Low Temp. Phys..

[20] Probst C, Wittig J (1977). Superconductivity of bcc barium under pressure. Phys. Rev. Lett..

[21] Mito M, Matsui H, Tsuruta K, Yamaguchi T, Nakamura K, Deguchi H, Shirakawa N, Adachi H, Yamasaki T, Iwaoka H, Ikoma Y, Horita Z (2016). Large enhancement of superconducting transition temperature in single-element superconducting rhenium by shear strain. Sci. Rep..

[22] Nishizaki T, Lee S, Horita Z, Sasaki T, Kobayashi N (2013). Superconducting properties in bulk nanostructured niobium prepared by high-pressure torsion. Physica C.

[23] Mito M, Kitamura Y, Tajiri T, Nakamura K, Shiraishi R, Ogata K, Deguchi H, Yamaguchi T, Takeshita N, Nishizaki T, Edalati K, Horita Z (2019). Hydrostatic pressure effects on superconducting transition of nanostructured niobium highly strained by high-pressure torsion. J. Appl. Phys..

[24] Mito M, Shigeoka S, Kondo H, Noumi N, Kitamura Y, Irie K, Nakamura K, Takagi S, Deguchi H, Tajiri T, Ishizuka M, Nishizaki T, Edalati K, Horita Z (2019). Hydrostatic compression effects on fifth-group element superconductors V, Nb, and Ta subjected to high-pressure torsion. Mater. Trans..

[25] Edalati K, Matsubara E, Horita Z (2009). Processing pure Ti by high-pressure torsion in wide range of pressures. Metall. Mater. Trans. A.

[26] Mito M, Shibayama K, Deguchi H, Tsuruta K, Tajiri T, Edalati K, Horita Z (2017). Contactless measurement of electrical conductivity for bulk nanostructured silver prepared by high-pressure torsion: A study of the dissipation process of giant strain. J. Appl. Phys..

[27] Ying J, Liu S, Lu Q, Wen X, Gui Z, Zhang Y, Wang X, Sun J, Chen X (2023). Record high 36 K transition temperature to the superconducting state of elemental scandium at a pressure of 260 GPa. Phys. Rev. Lett..

[28] Liu, X., Jiang, P., Wang, Y., Li, M., Li, N., Zhang, Q., Wang, Y., Li, Y-L., Yang, W. *T*_*c*_ up to 23.6 K and robust superconductivity in the transition metal δ-Ti phase at megabar pressure. *Phys. Rev*. *B* **105** (2022) 224511.

[29] Zhang C, He X, Liu C, Li Z, Lu K, Zhang S, Feng S, Wang X, Peng Y, Long Y, Yu R, Wang L, Prakapenka V, Chariton S, Li Q, Liu H, Chen C, Jin C (2022). Record high *T*_c_ element superconductivity achieved in titanium. Nature Commun..

[30] Sakata M, Nakamoto Y, Shimizu K, Matsuoka T, Ohish Y (2011). Superconducting state of Ca-VII below a critical temperature of 29 K at a pressure of 216 GPa. Phys. Rev. B.

[31] Smirnova NA, Levit VI, Pilyugin VI, Kuznetsov RI, Davydova LS, Sazonova VA (1986). Evolution of the structure of FCC single crystals upon large plastic deformations. Fiz Metal Metalloved.

[32] Harai Y, Ito Y, Horita Z (2008). High-pressure torsion using ring specimens. Scripta Mater..

[33] Zhilyaev AP, Langdon TG (2008). Using high-pressure torsion for metal processing: Fundamentals and applications. Prog. Mater. Sci..

[34] Edalati K, Horita Z (2016). A review on high-pressure torsion (HPT) from 1935 to 1988. Mater. Sci. Eng. A.

[35] McMillan WL (1968). Transition temperature of strong-coupled superconductors. Phys. Rev..

[36] Allen PB, Dynes RC (1975). Transition temperature of strong-coupled superconductors reanalyzed. Phys. Rev. B.

[37] Zhou D-W, Pu C-Y, Song H-Z, Li G-Q, Song J-F, Lu C, Bao G (2013). Superconducting properties of barium in three phases under high pressure from first principles. Chin. Phys. B.

[38] Takemura K (1994). High-pressure structural study of barium to 90 GPa. Phys. Rev. B.

[39] Edalati K, Miresmaeili R, Horita Z, Kanayama H, Pippan R (2011). Significance of temperature increase in processing by high pressure torsion. Mater. Sci. Eng. A.

[40] Edalati K, Daio T, Arita M, Lee S, Horita Z, Togo A, Tanaka I (2014). High-pressure torsion of titanium at cryogenic and room temperatures: Grain size effect on allotropic phase transformations. Acta Mater..

[41] Giannozzi, P., Baroni, S., Bonini, N., Calandra, M., Car, R., Cavazzoni, C., Ceresoli, D., do L Chiarotti, G., Cococcioni, M., Dabo, I., Corso, A. D., de Gironcoli, S., Fabris, S., Fratesi, G., Gebauer, R., Gerstmann, U., Gougoussis, C., Kokalj, A., Lazzeri, M., Martin-Samos, L., Marzari, N., Mauri, F., Mazzarello, R., Paolini, S., Pasquarello, A., Paulatto, L., Sbraccia, C., Scandolo, S., Sclauzero, G., Seitsonen, A. P., Smogunov, A., Umari, P., & Wentzcovitch, R. M. QUANTUM ESPRESSO: A modular and open-source software project for quantum simulations of materials. *J. Phys.: Condens. Matter* **21**, 395502 (2009). 10.1088/0953-8984/21/39/395502 21832390

[42] Giannozzi, P., Andreussi, O., Brumme, T., Bunau, O., Nardelli, M. B., Calandra, M., Car, R., Cavazzoni, C., Ceresoli, D., Cococcioni, M., Colonna, N., Carnimeo, I., Dal Corso, A., de Gironcoli, S., Delugas, P., DiStasio, R. A., Jr, Ferretti, A., Floris, A., Fratesi, G., Fugallo, G., Gebauer, R., Gerstmann, U., Giustino, F., Gorni, T., Jia, J., Kawamura, M., Ko, H.-Y., Kokalj, A., Kucukbenli, E., Lazzeri, M., Marsili, M., Marzari, N., Mauri, F., Nguyen, N. L., Nguyen, H.-V., Otero-de-la Roza, A., Paulatto, L., Ponce, S., Rocca, D., Sabatini, R., Santra, B., Schlipf, M., Seitsonen, A. P., Smogunov, A., Timrov, I., Thonhauser, T., Umari, P., Vast, N., Wu, X., & Baroni, S. Advanced capabilities for materials modelling with Quantum ESPRESSO. *J. Phys.: Condens. Matter* **29**, 465901 (2017). 10.1088/1361-648X/aa8f79 29064822

[43] Perdew JP, Burke K, Ernzerhof M (1996). Generalized gradient approximation made simple. Phys. Rev. Lett..

[44] Hamann DR (2013). Optimized norm-conserving Vanderbilt pseudopotentials. Phys. Rev. B.

[45] Setten, M. J., Giantomassi, M., Bousquet, E., Verstraete, M.J., Hamann, D. R., Gonze, X., & Rignanese. G.-M. The PseudoDojo: Training and grading a 85 element optimized norm-conserving pseudopotential table. *Comput. Phys. Commun.* **226**, 39 (2018).

[46] Methfessel M, Paxton AT (1989). High-precision sampling for Brillouin-zone integration in metals. Phys. Rev. B.

[47] Baroni S, Giannozzi P, Testa A (1987). Green’s-function approach to linear response in solids. Phys. Rev. Lett..

[48] Giannozzi P, de Gironcoli S, Pavone P, Baroni S (1991). Ab initio calculation of phonon dispersions in semiconductors. Phys. Rev. B.

