# Supplementary material for: Superconductivity of barium with highest transition temperatures in metallic materials at ambient pressure
Source: Sci Rep. 2024 Jan 16;14:965. doi: 10.1038/s41598-023-50940-5 (PMC10791626; doi:10.1038/s41598-023-50940-5)
Supplement: Supplementary file 1 — Supplementary Information. [file 41598_2023_50940_MOESM1_ESM.doc]

**<Supplementary Information: Extended Data>**

Superconductivity of Barium with highest transition temperatures in metallic materials at ambient pressure

Masaki Mito^1^, Hiroki Tsuji^1^, Takayuki Tajiri^2^, Kazuma Nakamura^1^, Yongpeng Tang^1^, and Zenji Horita^1^

^1^ Graduate School of Engineering, Kyushu Institute of Technology, Kitakyushu 804-8550, Japan

^2^ Faculty of Science, Fukuoka University, Fukuoka 814-0180, Japan

**List of Figures**

**Extended Data Fig. 1** Overview of HPT

**Extended Data Fig. 2** Summary of previous hydrostatic pressure experiments.

**Extended Data Fig. 3** Magnetic data for HPT-Ba (samples 2 and 4) at *P*_HPT_ = 6 GPa and *N* = 10 at room temperature.

**Extended Data Fig. 4** Reflection type of XRD pattern of HPT-Ba at *P*_HPT_ = 6 GPa.

**Extended Data Fig. 5** Magnetic data for HPT-Ba (Sample No. 9–12) at *P*_HPT_ = 12, 15, 18, and 24 GPa and *N* = 10 in liquid nitrogen.

**Extended Data Fig. 6** Reflection type of the XRD experiment for HPT-Ba (Sample No. 9) at *P*_HPT_ = 12 GPa and *N* = 10 in liquid nitrogen

**Extended Data Fig. 7** Reflection type of the XRD experiment for HPT-Ba (Sample No. 12) at *P*_HPT_ = 21 GPa and *N* = 10 in liquid nitrogen

**Extended Data Fig. 8** Reflection type of XRD experiment for HPT-Ba (Sample No. 13) at *P*_HPT_ = 24 GPa and *N* = 10 in liquid nitrogen.

**Extended Data Fig. 9** Thrubeam type XRD experiment for HPT-Ba (Sample No. 9) at *P*_HPT_ = 12 GPa and *N* = 10 in liquid nitrogen.

**Extended Data Fig. 10** Thrubeam type XRD experiment for HPT-Ba (Sample No. 10) at *P*_HPT_ = 15 GPa and *N* = 10 in liquid nitrogen

**Extended Data Fig. 11** Thrubeam type XRD experiment for HPT-Ba (Sample No. 11) at *P*_HPT_ = 18 GPa and *N* = 10 in liquid nitrogen.

**Extended Data Fig. 12** Thrubeam type XRD experiment for HPT-Ba (Sample No. 12) at *P*_HPT_ = 21 GPa and *N* = 10 in liquid nitrogen.

**Extended Data Fig. 13** Thrubeam type of XRD experiment for HPT-Ba (Sample No. 13) at *P*_HPT_ = 24 GPa and *N* = 10 in liquid nitrogen.

**Extended Data Fig. 14** Change in atomic volume under hydrostatic pressure.

**List of Tables**

**Extended Data Table 1** Summary of the experimental results.

**Extended Data Table 2** Summary of the reflection types of the XRD analysis

**Extended Data Table 3** Summary of the thrubeam types of XRD analysis at SPring-8.


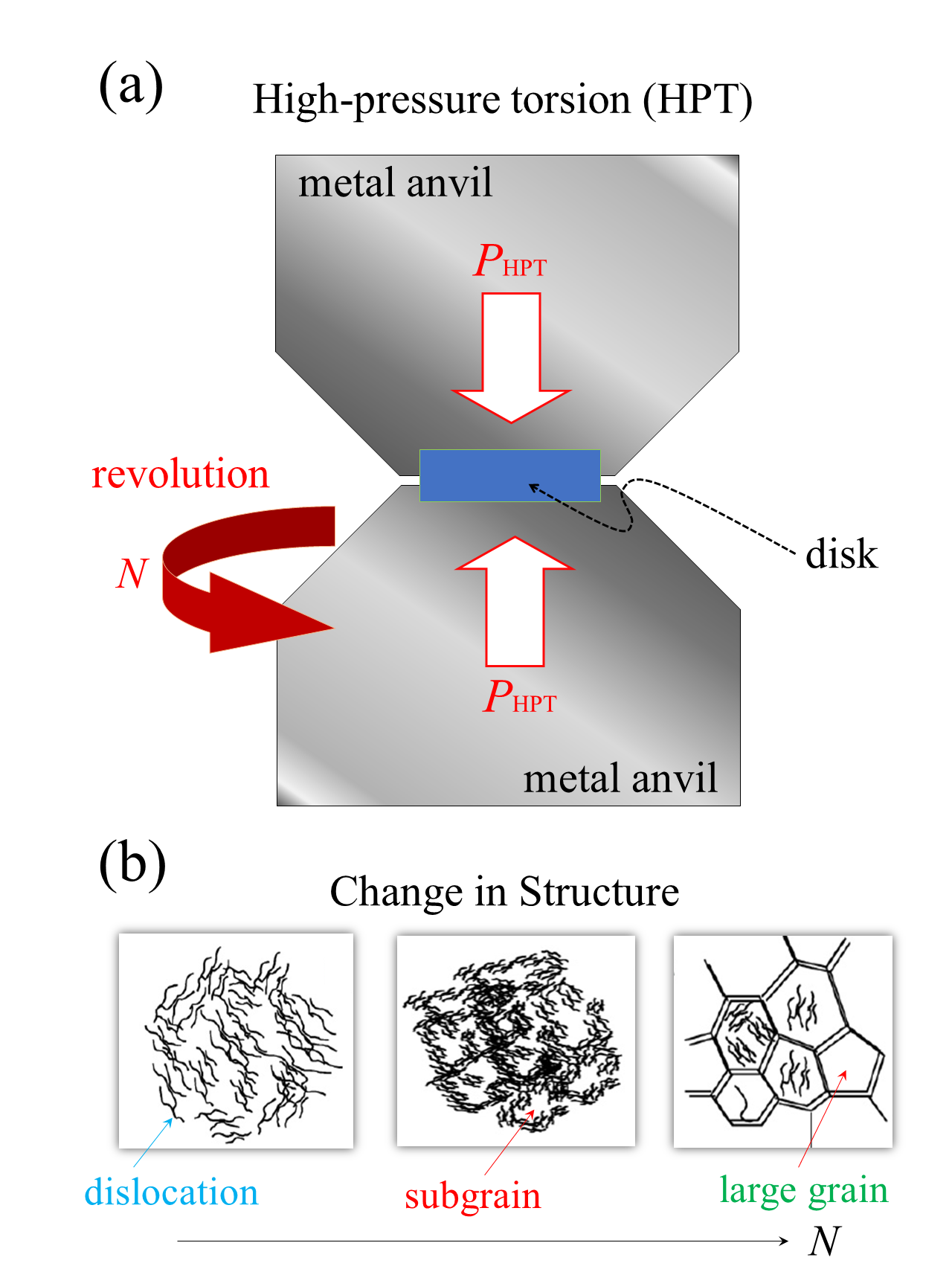


Extended Data Fig. 1 Overview of high-pressure torsion (HPT).

(a) Overview of HPT setup. After uniaxial compression was applied using oppositely facing metal anvils, a shear strain was applied by rotating the lower anvil. (b) Change in structural grains due to HPT. As *N* increased, dislocations accumulated, and the grain size decreased. Under sufficient strain accumulation, the reduction in the grain size was balanced by recrystallization.


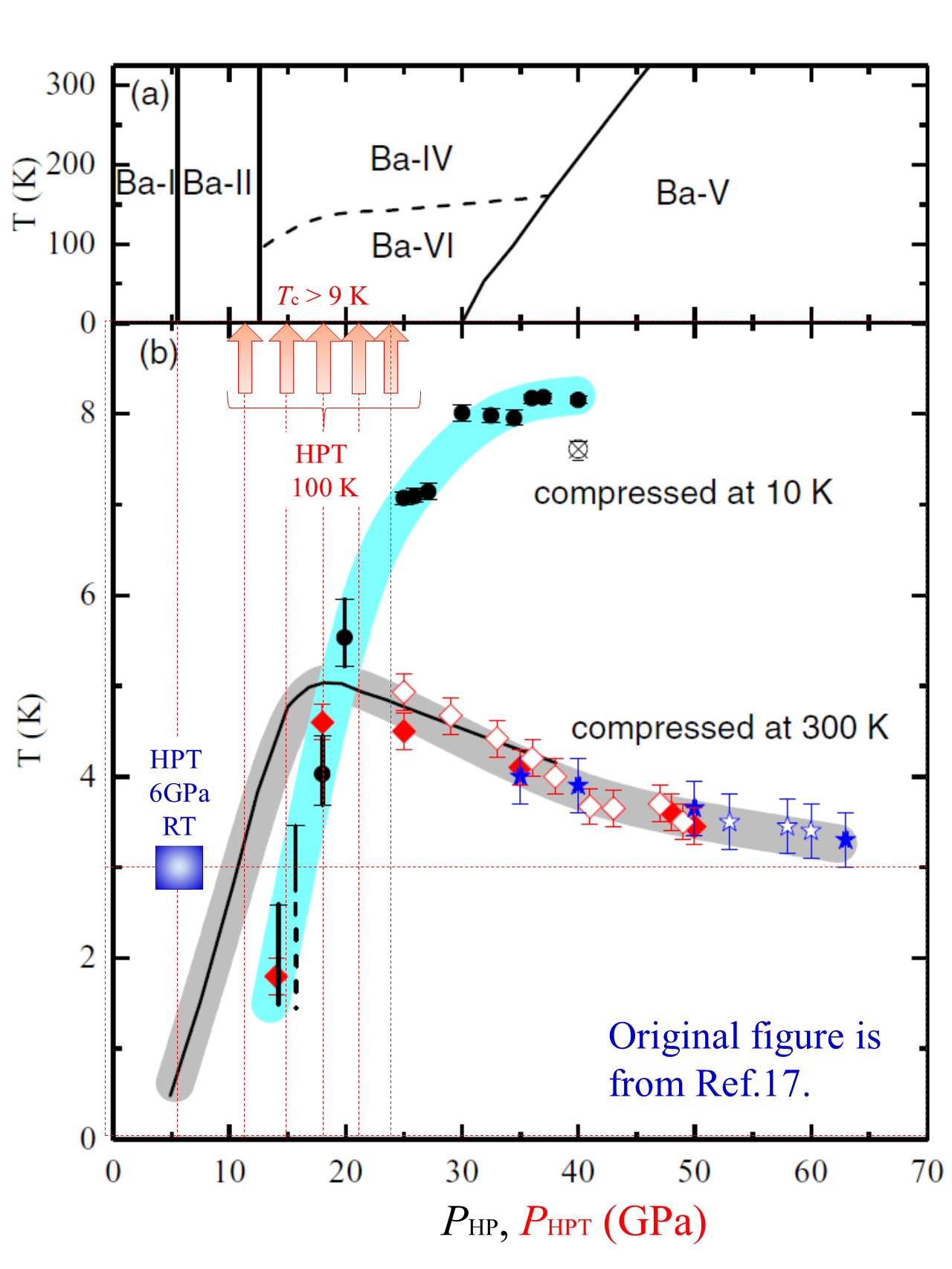


Extended Data Fig. 2 Previous studies on hydrostatic contraction of Ba.

Present results of *T*_c_ as a function of *P*_HPT_ is plotted on the literature figure on *T*_c_ as a function of hydrostatic pressure, *P*_HP_ ^17^. The gray curve represents the change in *T*_c_ for hydrostatic contraction at 300 K, and the light blue curve represents the change in *T*_c_ at 10 K.


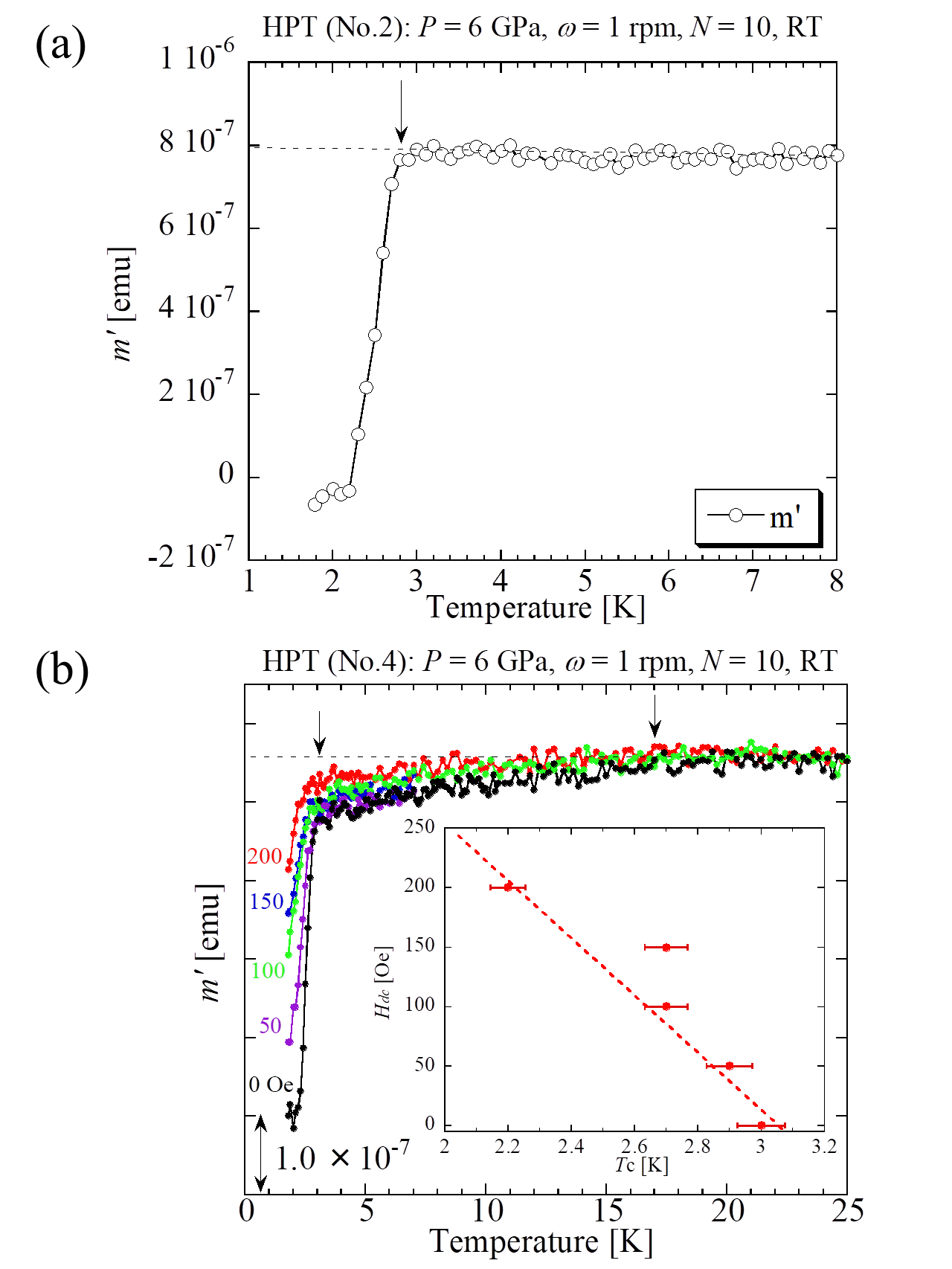


Extended Data Fig. 3 Magnetic data of HPT-Ba at *P*_HPT_ = 6 GPa.

(a) In material No. 2, the superconducting signal was observed to have almost the same volume fraction as that in material No. 1. The *T*_c_ at *H*_dc_ = 0 was 2.8 K. (b) The No. 4 material also exhibited the *H*_dc_ dependence of the diamagnetic signal similar to No.1


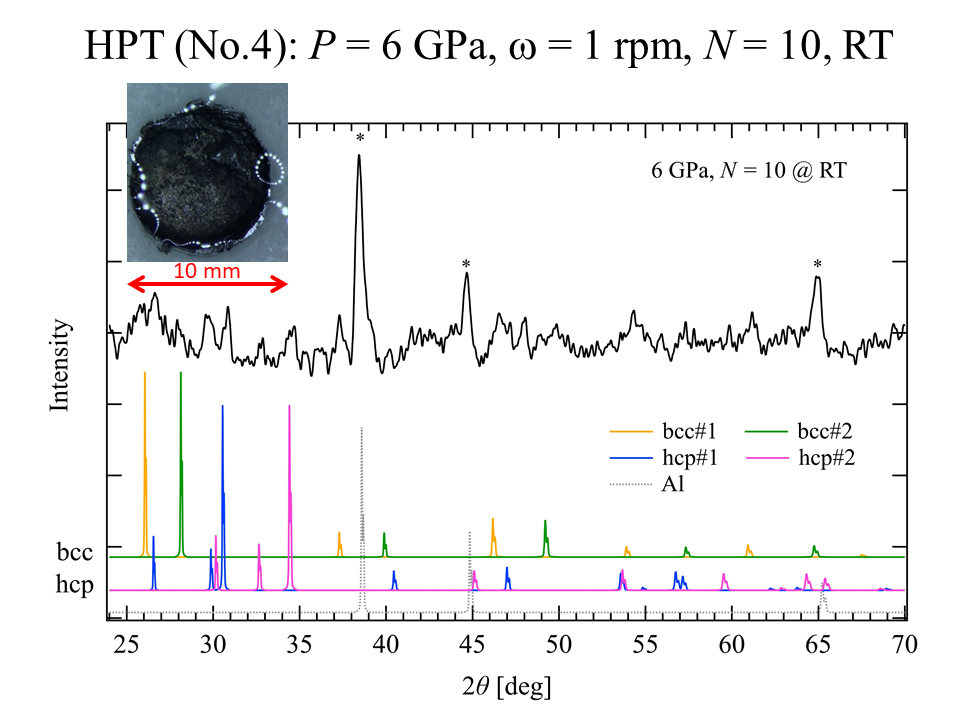


Extended Data Fig. 4 XRD pattern of HPT-Ba at *P*_HPT_ = 6 GPa.

The XRD patterns of sample No. 4 with *T*_c_ = 3.0 K. This indicates the existence of both bcc and hcp phases. For No. 4, the anomalies due to Al in the sample cell also appear, in addition to bcc #1 with *a* = 4.786 Å, bcc #2 with *a* = 4.636 Å, hcp #1 with *a* = 3.854 Å and *c* = 5.963 Å, and hcp #2 with *a* = 3.422 Å and *c* = 5.434 Å. This result suggests that HPT stabilizes the high-pressure phase, even at ambient pressure after the HPT processing. The detailed lattice parameters are summarized in Extended Data Table 2.


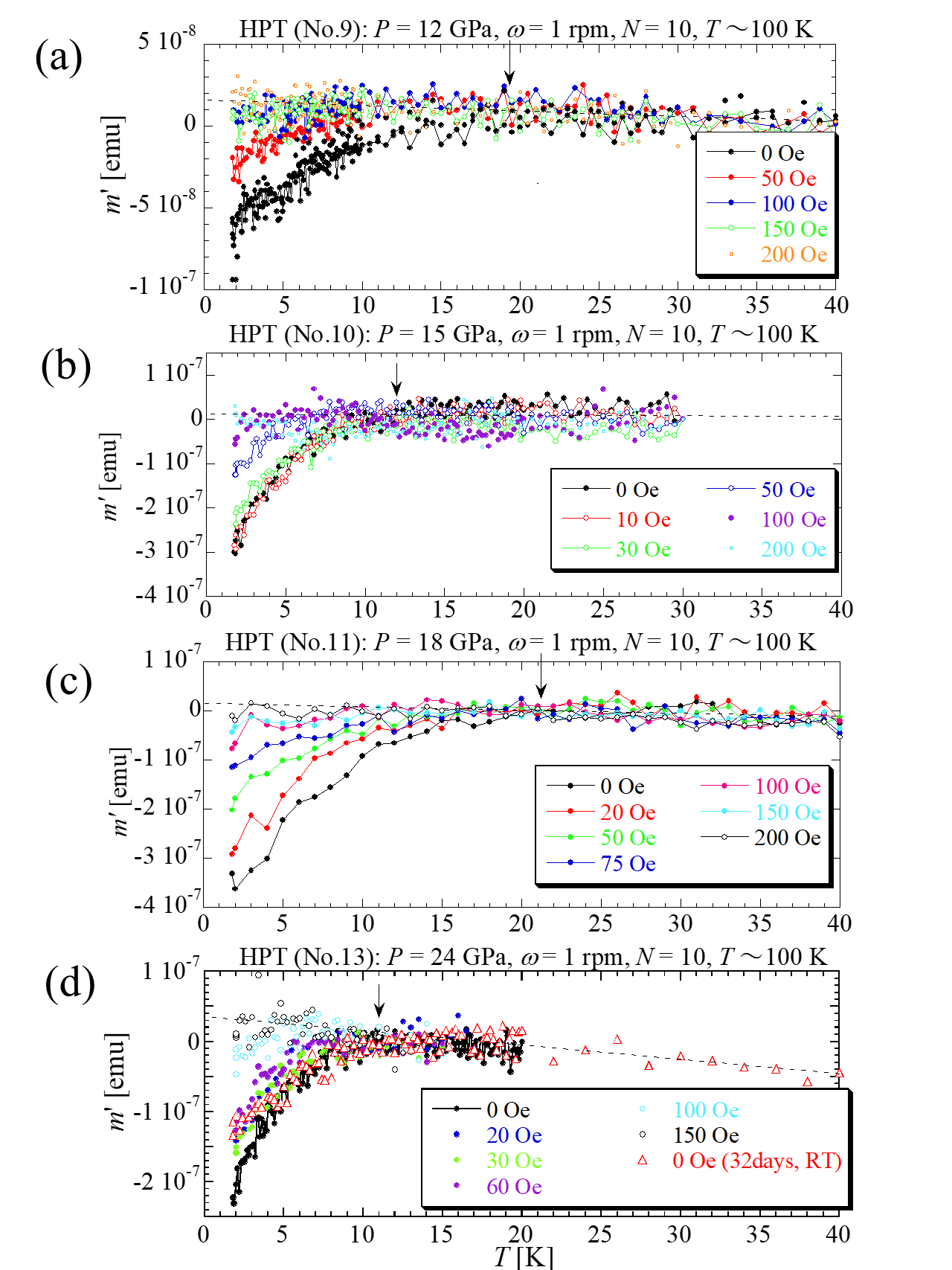


Extended Data Fig. 5 Temperature dependence of the AC magnetization for HPT-Ba at *P*_HPT_ = 12 (a), 15 (b), 18 (c), and 24 GPa (d).

In (d), a diamagnetic signal of approximately 75 % remains even after 32 days at room temperature.


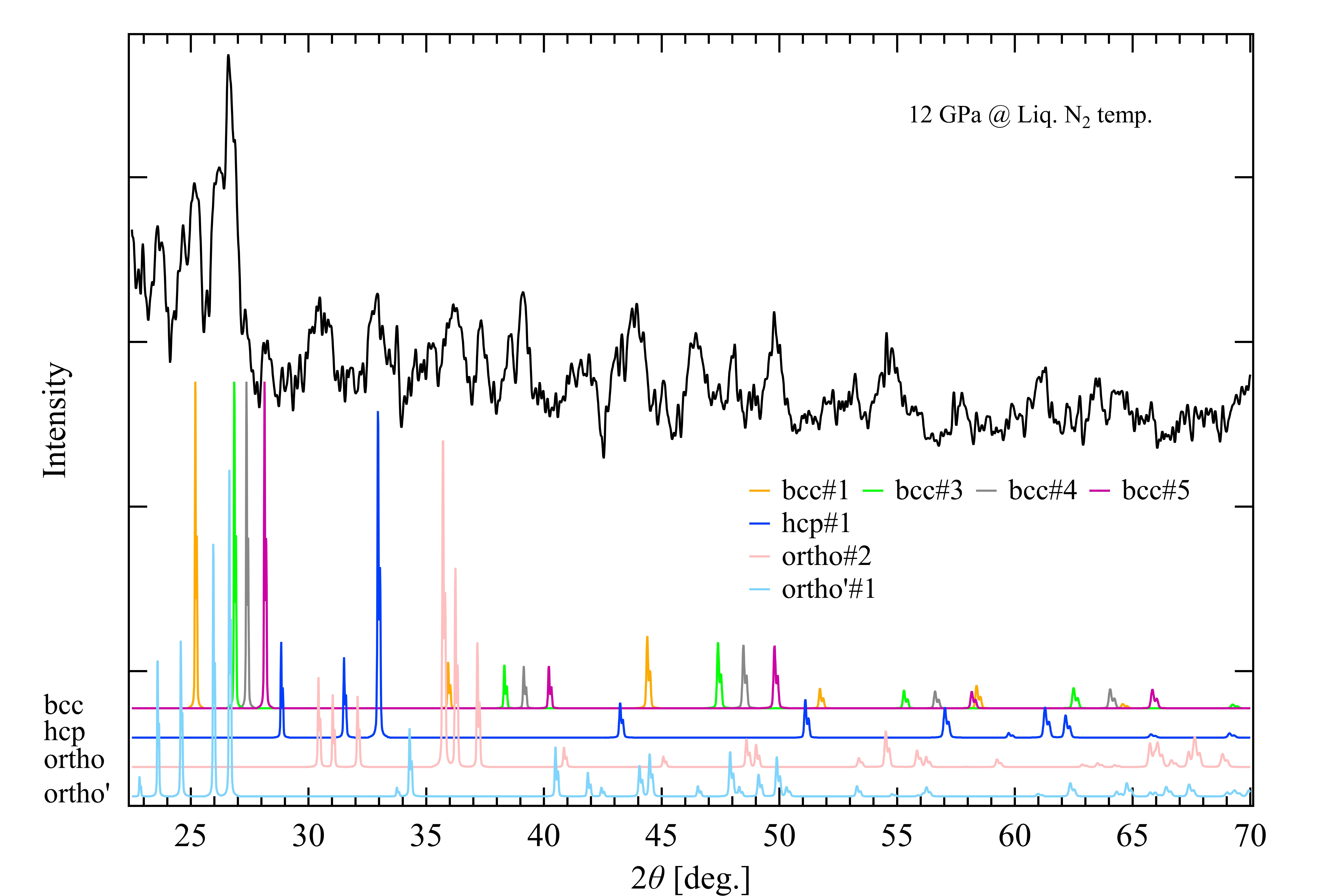


Extended Data Fig. 6 XRD pattern of HPT-Ba at *P*_HPT_ = 12 GPa.

XRD pattern of No. 9 with *T*_c_ = 8.0 K. Sample 9 was subjected to HPT in liquid nitrogen. Two orthorhombic states appeared along with four bcc and one hcp. The detailed lattice parameters are summarized in Extended Data Table 2.


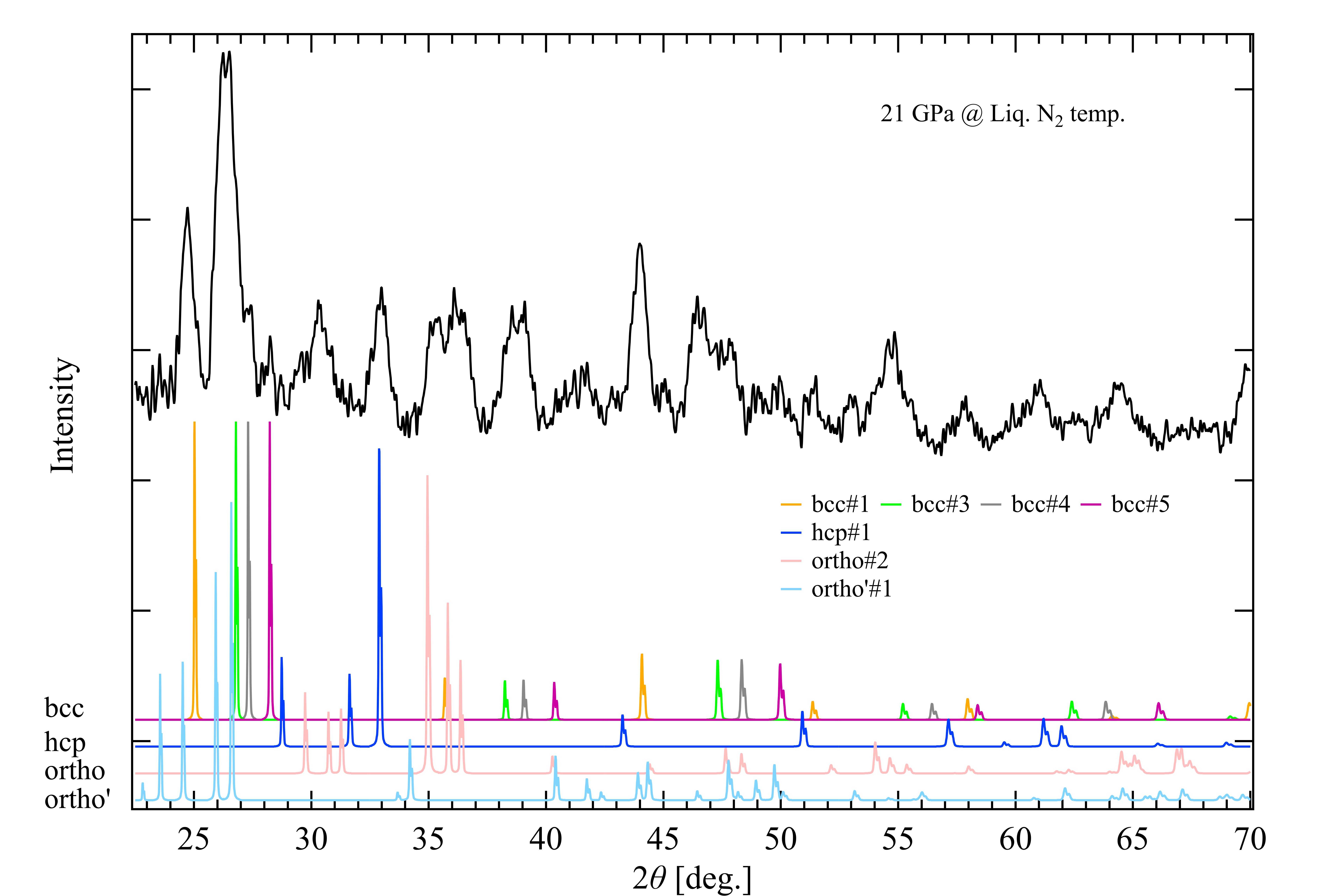


Extended Data Fig. 7 XRD pattern of HPT-Ba at *P*_HPT_ = 21 GPa.

XRD pattern of No. 12 with *T*_c_ = 24 K. Sample 12 was subjected to HPT in liquid nitrogen. Two orthorhombic states appeared along with four bcc and one hcp. The detailed lattice parameters are summarized in Extended Data Table 2.


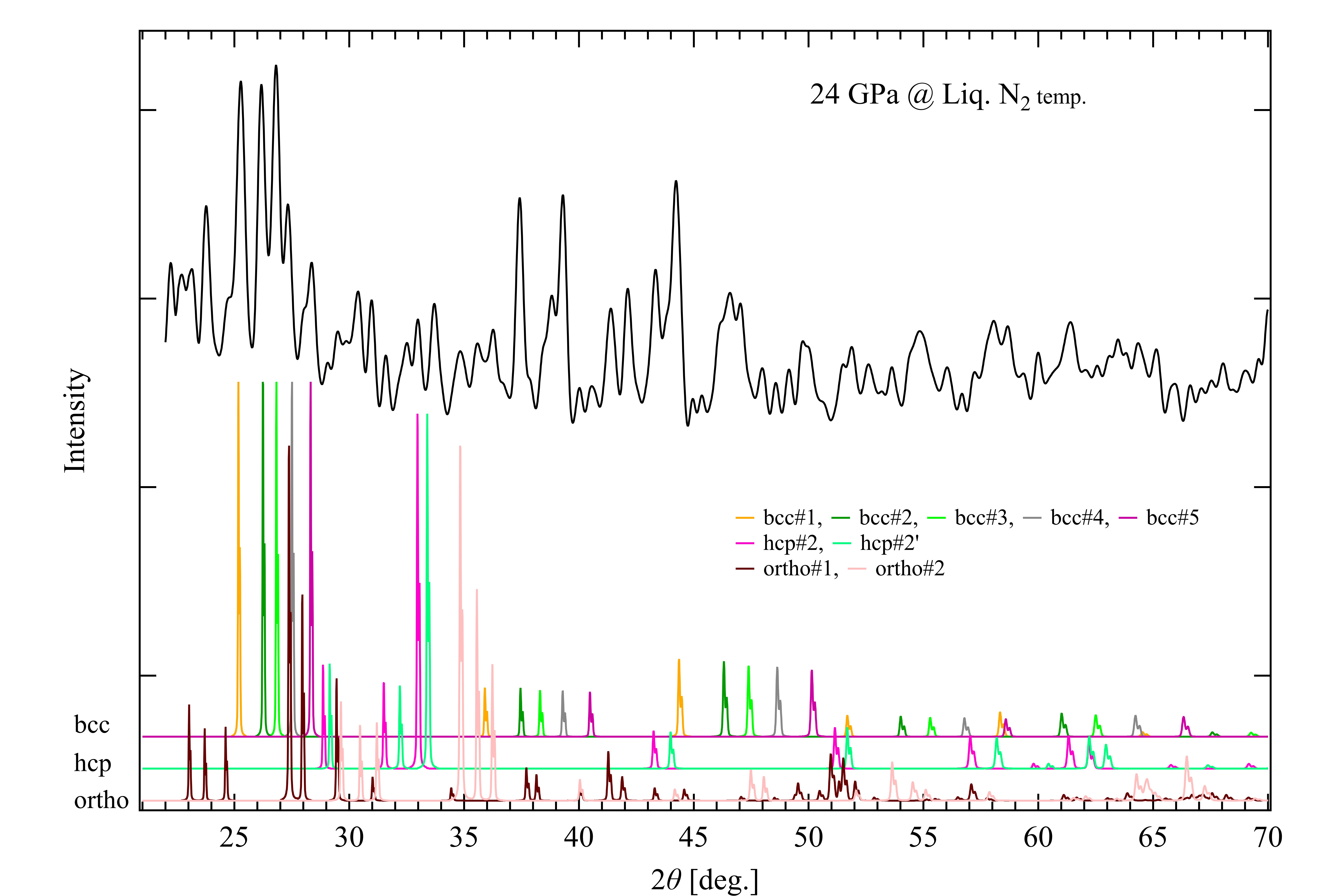


Extended Data Fig. 8 XRD pattern of HPT-Ba at *P*_HPT_ =24 GPa.

XRD pattern of No. 13 at *T*_c_ of 11 K. Sample 13 was subjected to HPT in liquid nitrogen. Two orthorhombic states appeared, along with five bcc and two hcp states. The detailed lattice parameters are summarized in Extended Data Table 2.


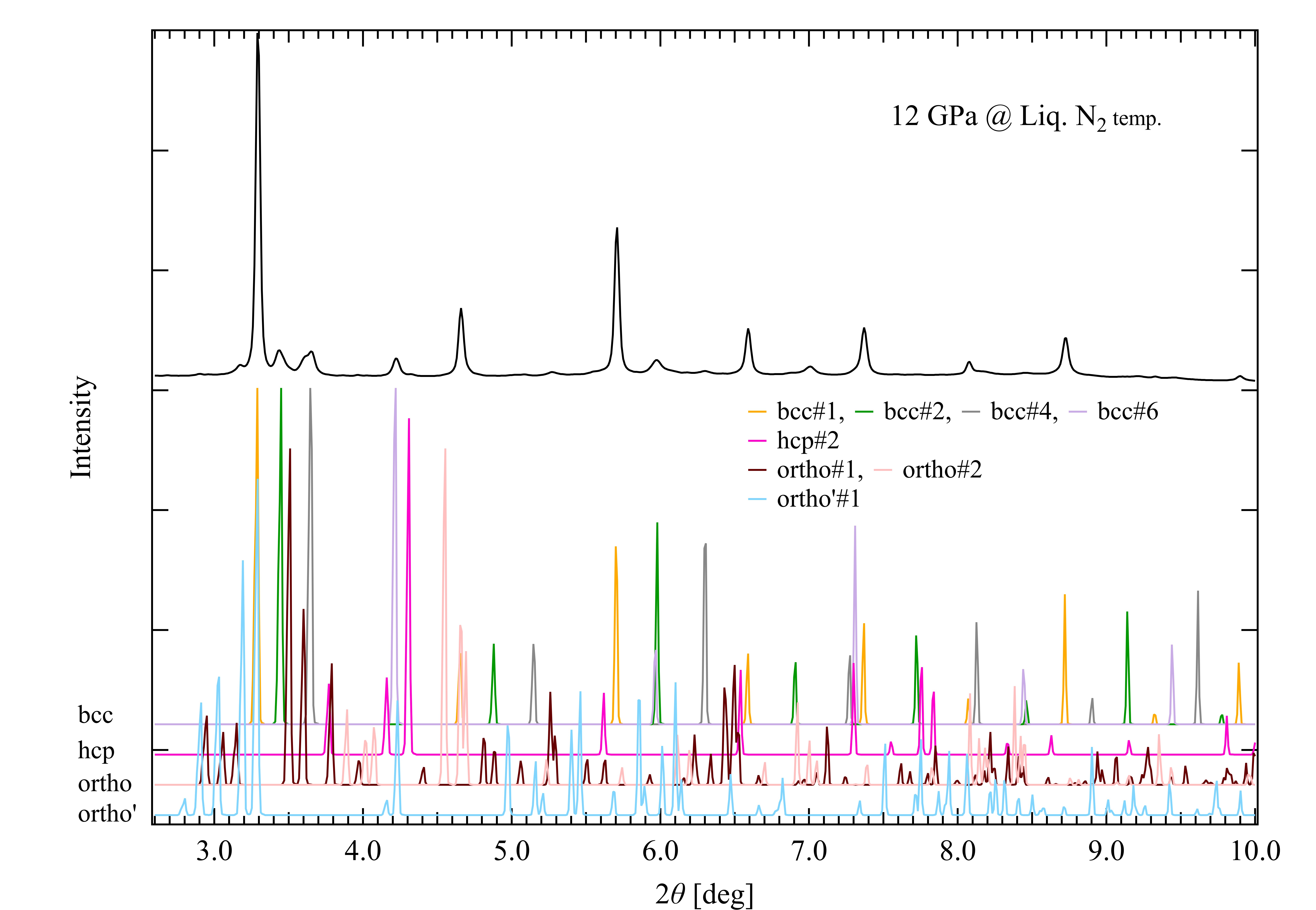


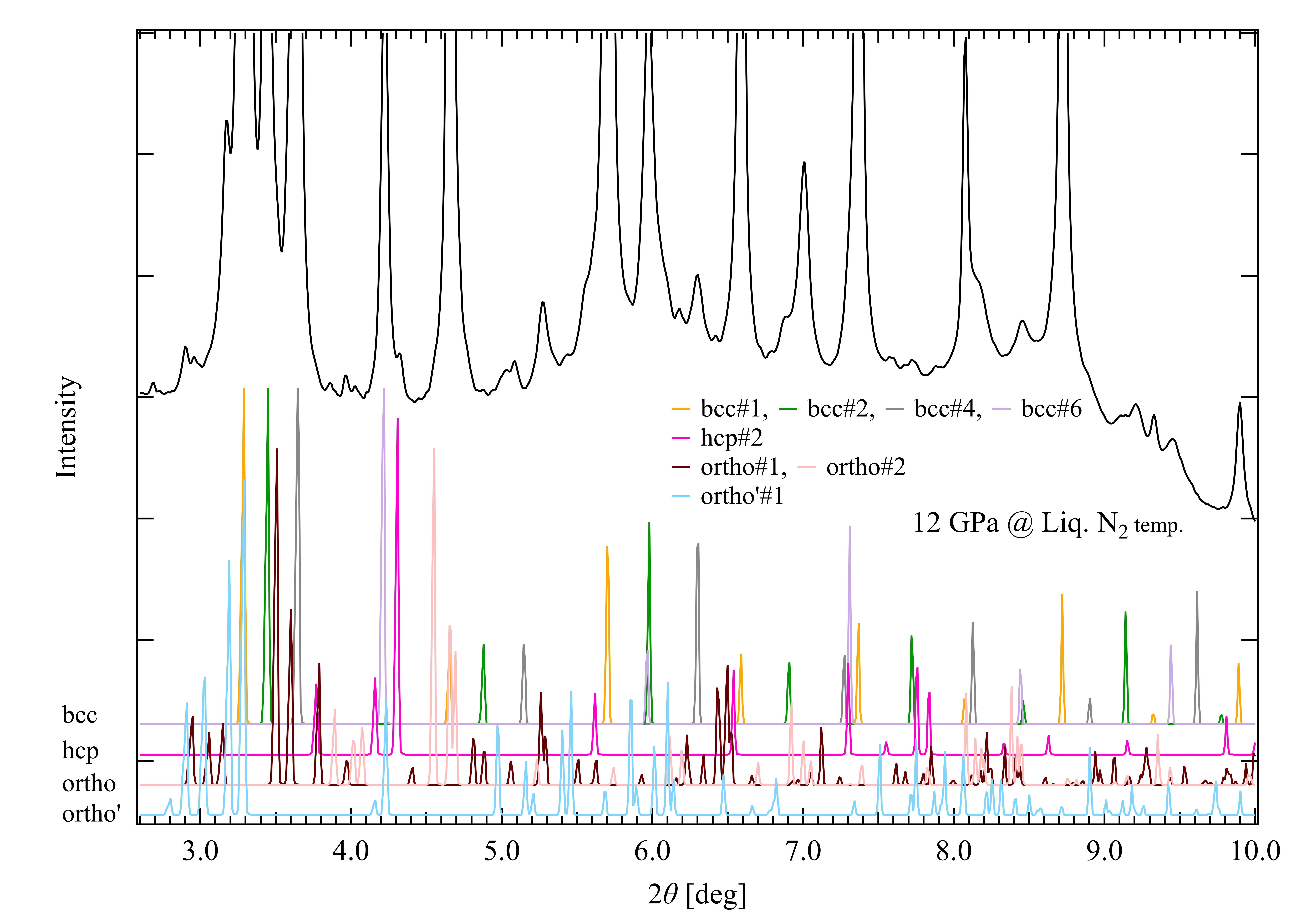


Extended Data Fig. 9 XRD patterns of thrubeam method for HPT-Ba (No. 9) at *P*_HPT_ = 12 GPa.

Detailed lattice parameters are summarized in Extended Data Table 3.


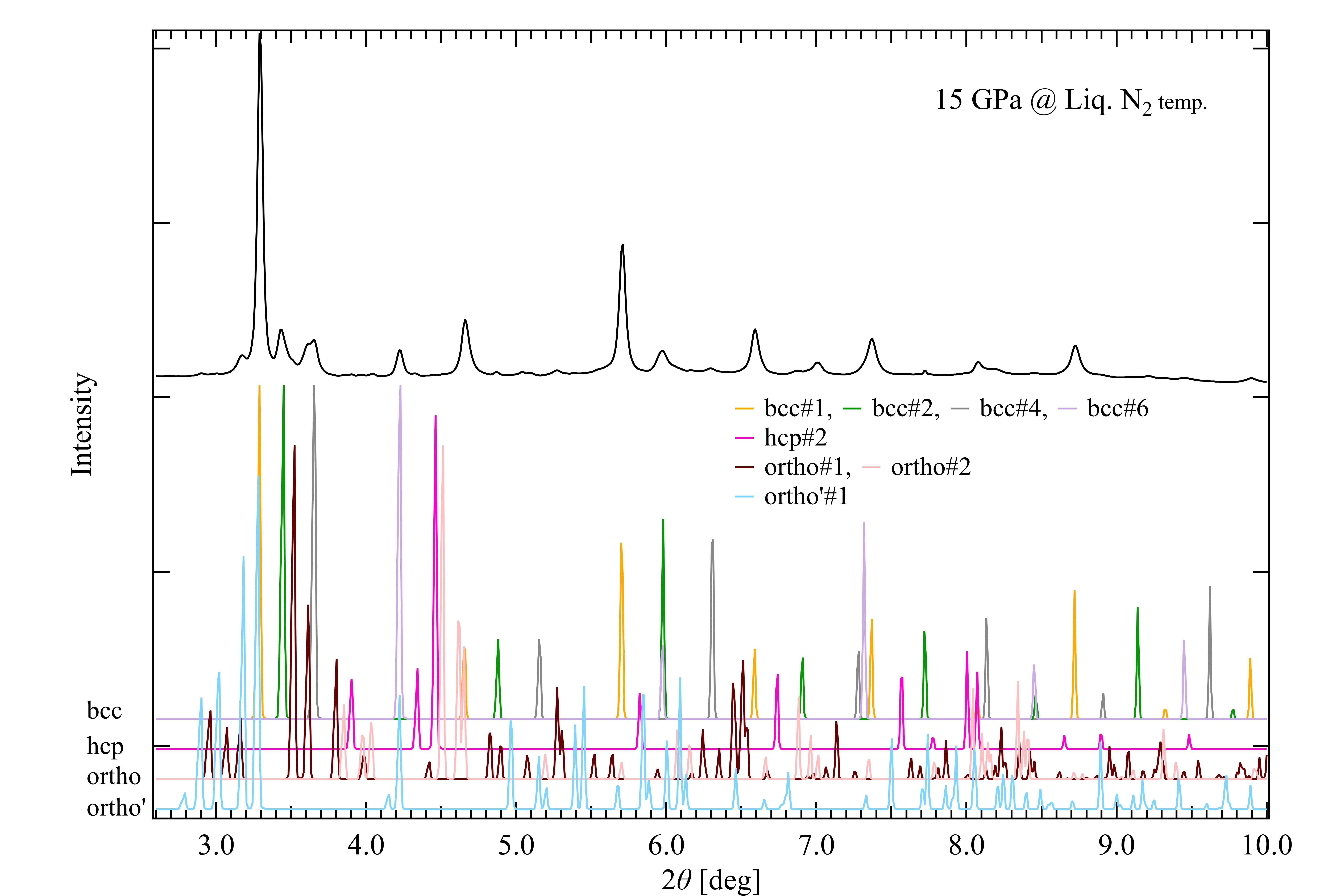


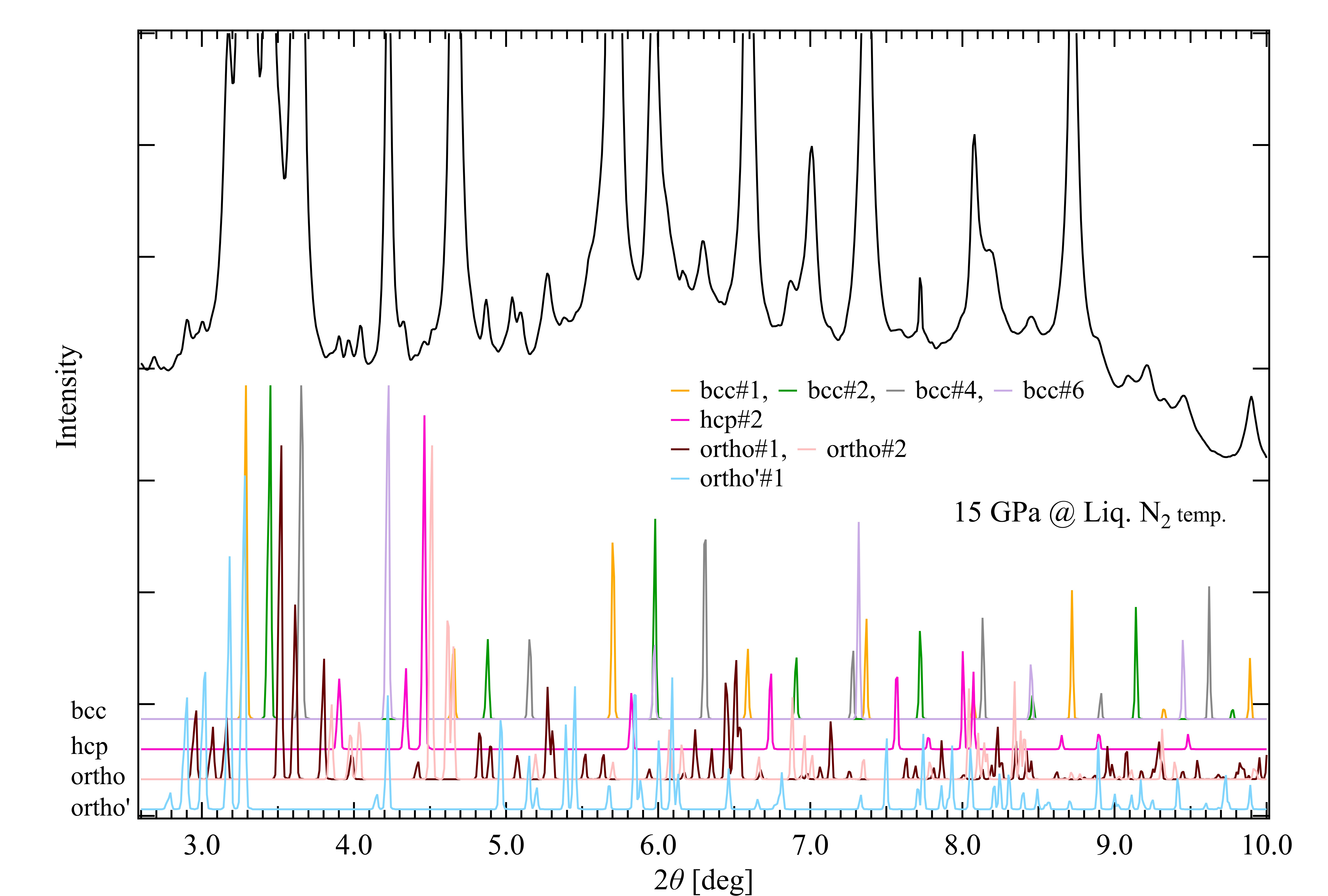


Extended Data Fig. 10 XRD patterns of thrubeam method for HPT-Ba (No. 10) at *P*_HPT_ = 15 GPa.

Detailed lattice parameters are summarized in Extended Data Table 3.


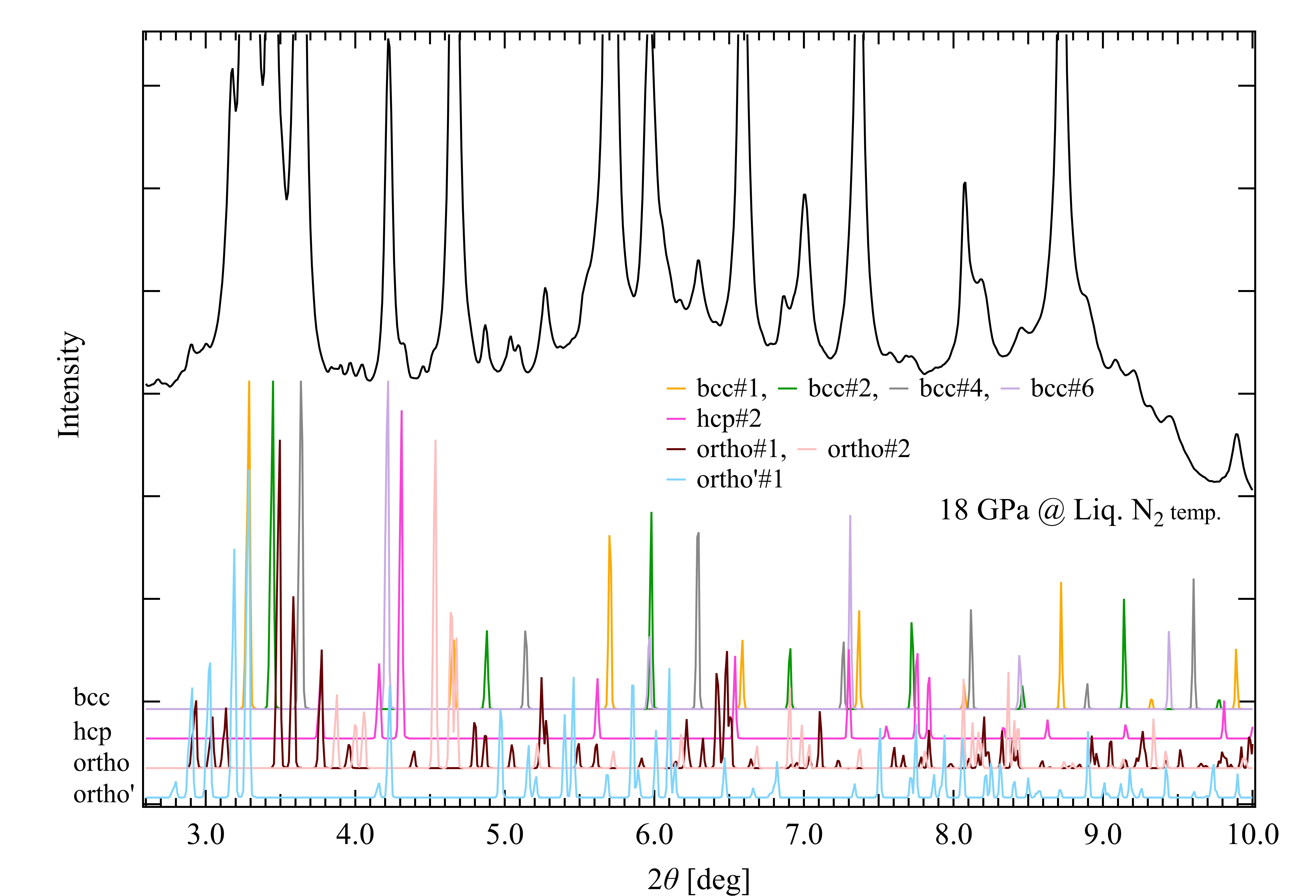

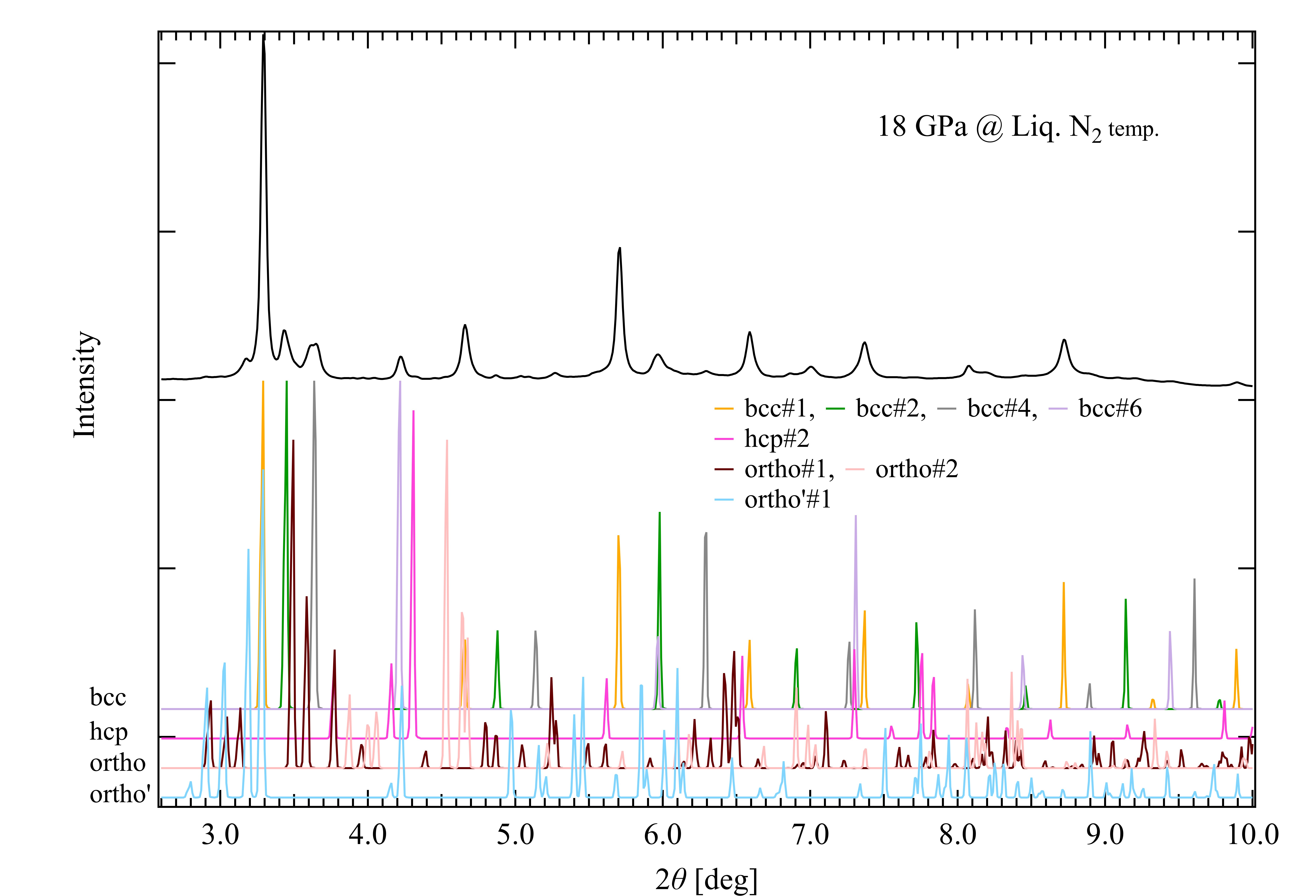


Extended Data Fig. 11 XRD patterns of thrubeam method for HPT-Ba (No. 11) at *P*_HPT_ = 18 GPa.

Detailed lattice parameters are summarized in Extended Data Table 3.


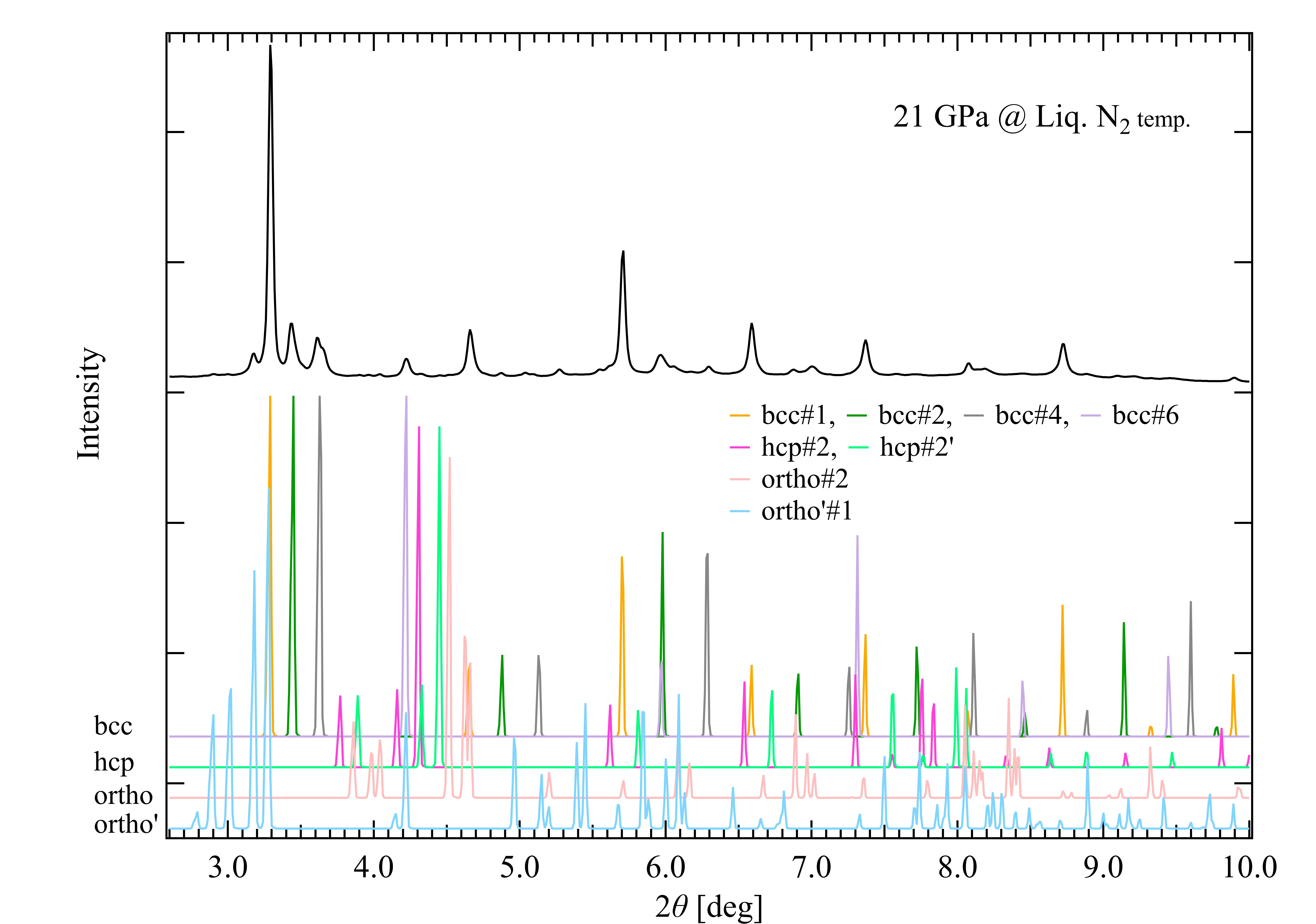

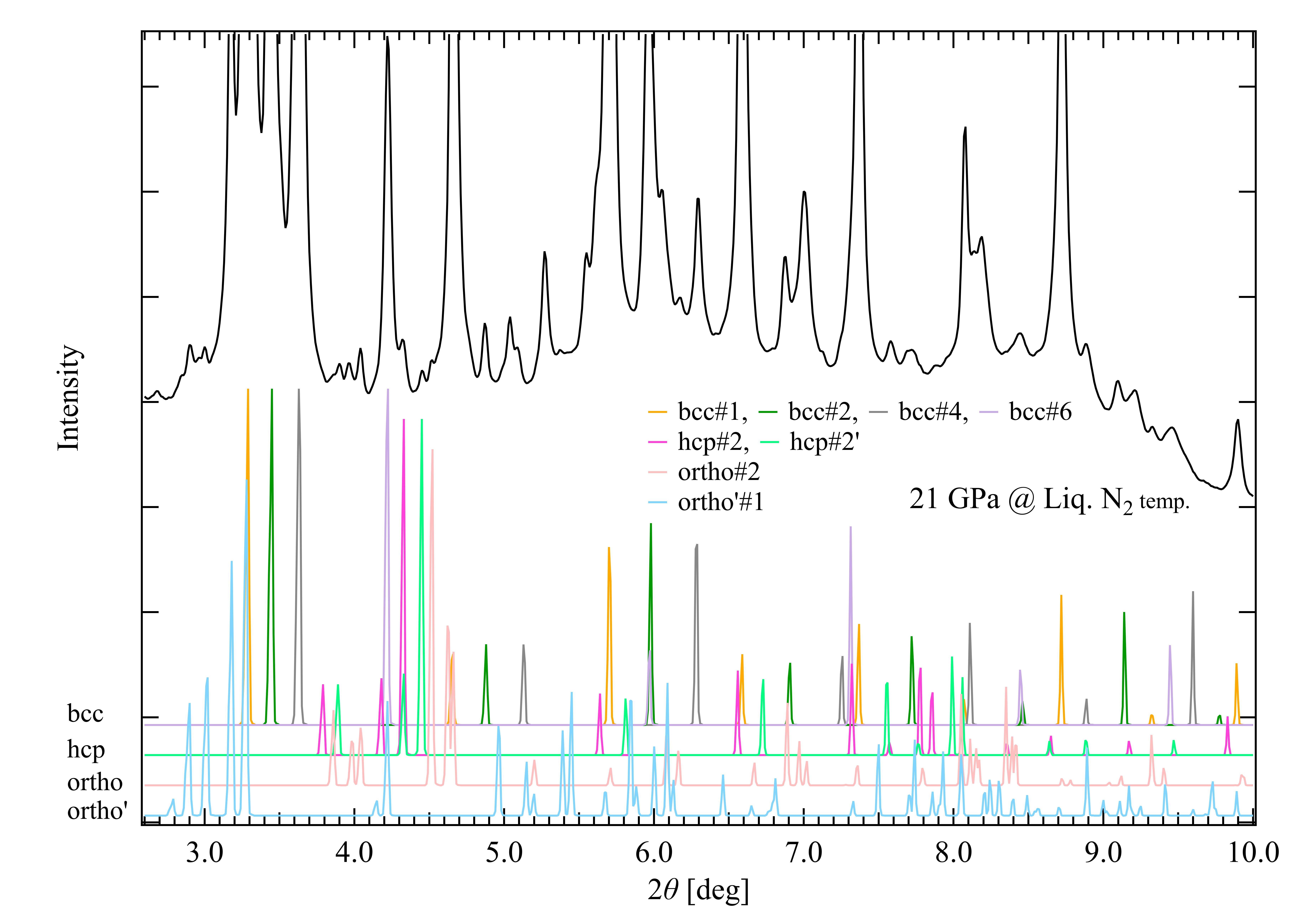


Extended Data Fig. 12 XRD patterns of thrubeam method for HPT-Ba (No. 12) at *P*_HPT_ = 21 GPa.

Detailed lattice parameters are summarized in Extended Data Table 3.


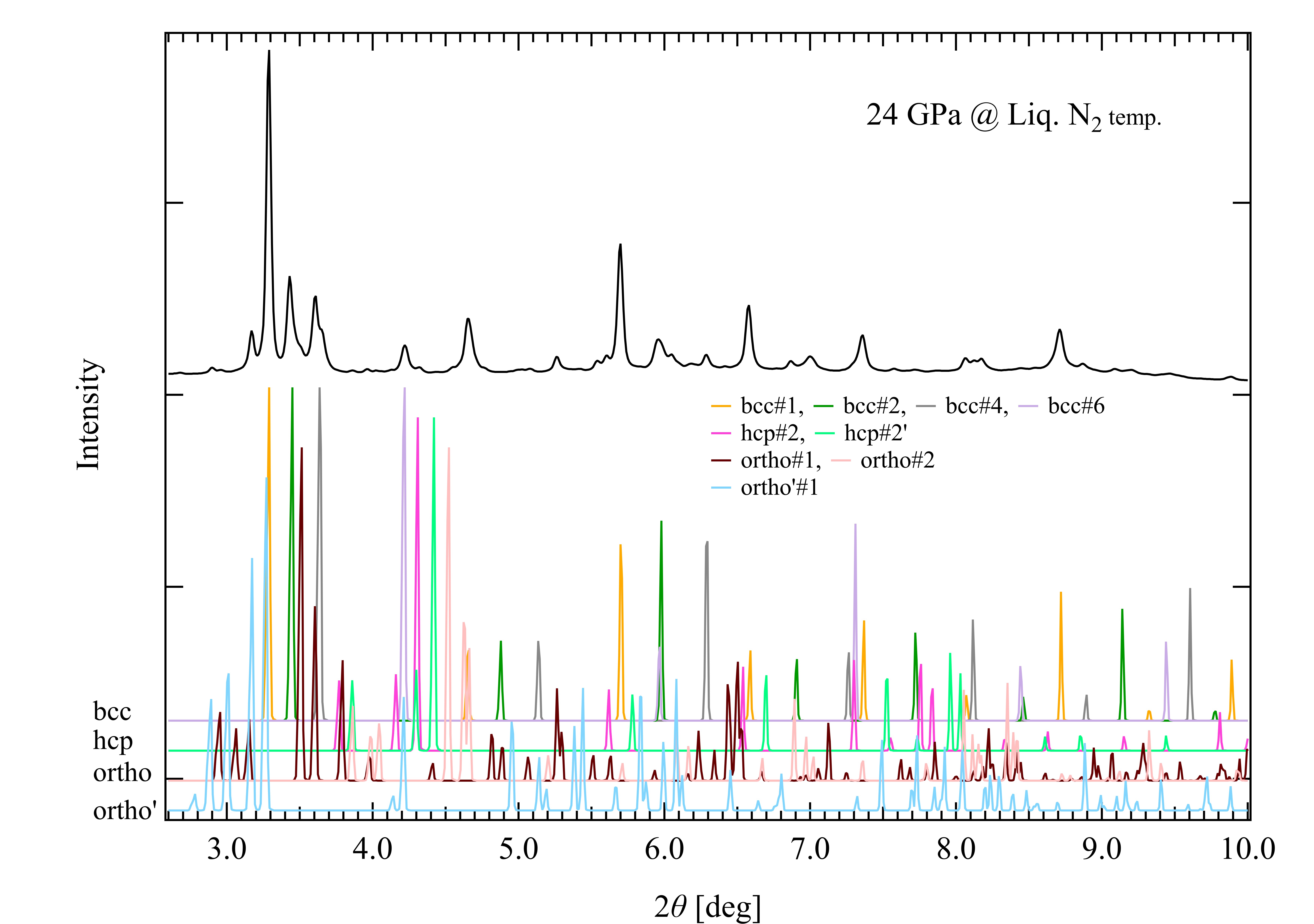


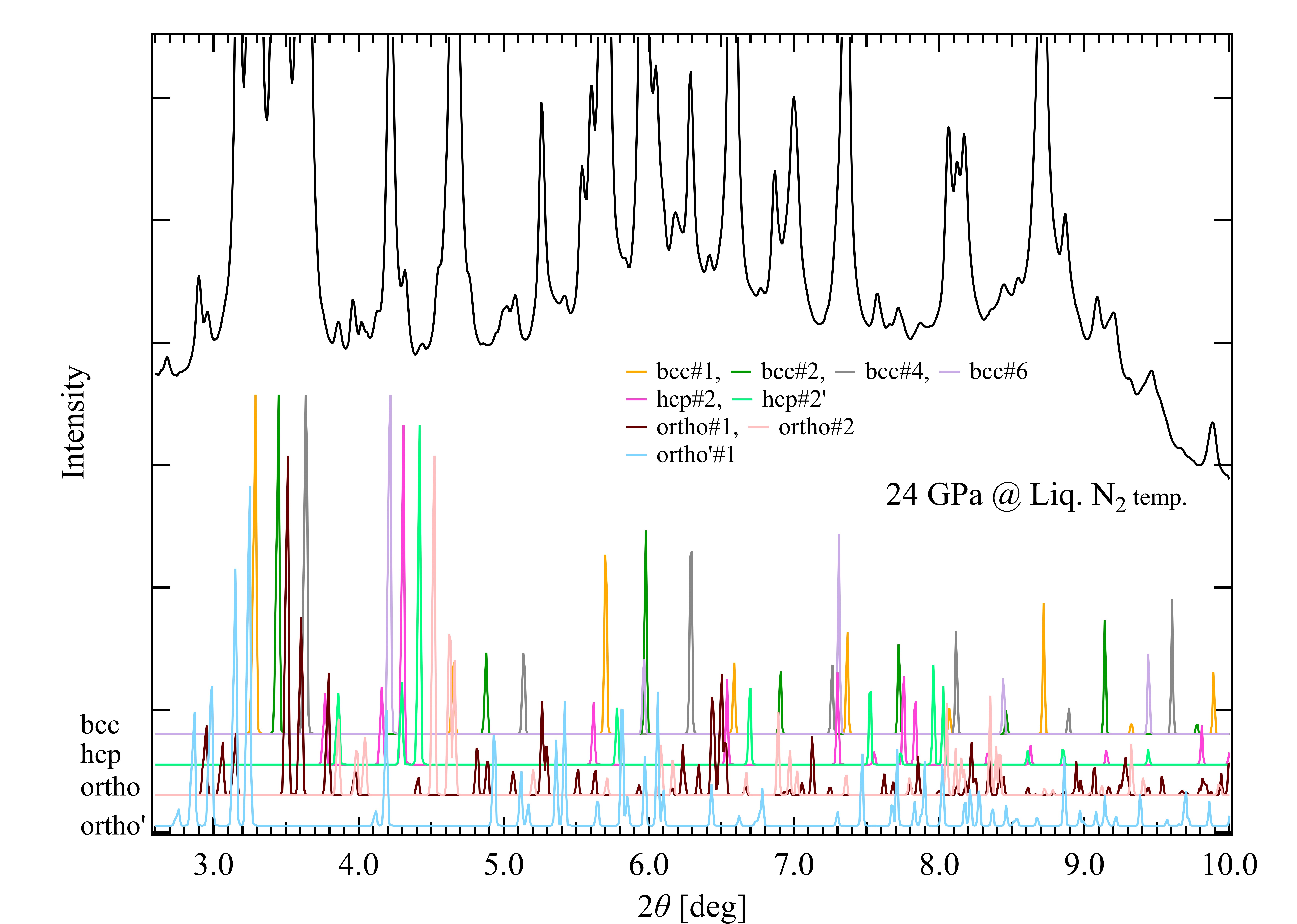


Extended Data Fig. 13 XRD patterns of the thrubeam method for HPT-Ba

(No. 13) at *P*_HPT_ = 24 GPa.

Detailed lattice parameters are summarized in Extended Data Table 3.


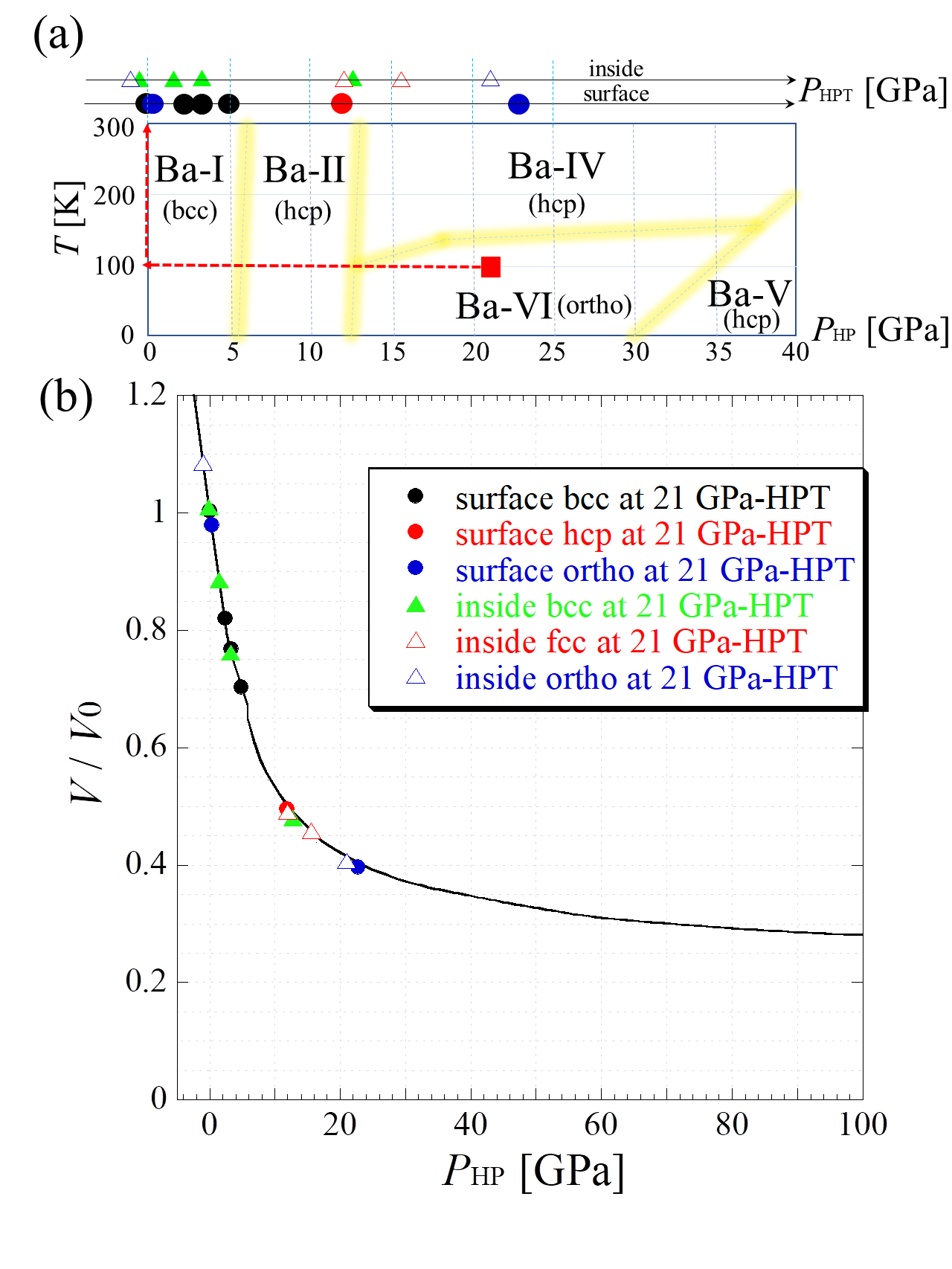


Extended Data Fig. 14 Change in atomic volume under hydrostatic pressure.

(a) shows the locations of the crystal structures of specimen No.12 (HPT-processed sample for *P*_HPT_ = 21 GPa at 100K) in the temperature-pressure phase diagram. The cases of the surface and inside are depicted together. (b) shows the location of the crystal structures of specimen No. 12 in the graphics of the atomic volume as a function of pressure ^38^. The cases of the surface and inside are depicted together.

Extended Data Table 1 Summary of the experimental results.

Conditions for Ba sample preparation using HPT RT: room temperature. *V*_SC_ is the volume fraction of the superconducting (SC) state. NE represents "non-estimated".

Extended Data Table 2 Summary of the reflection type of the XRD analyses.


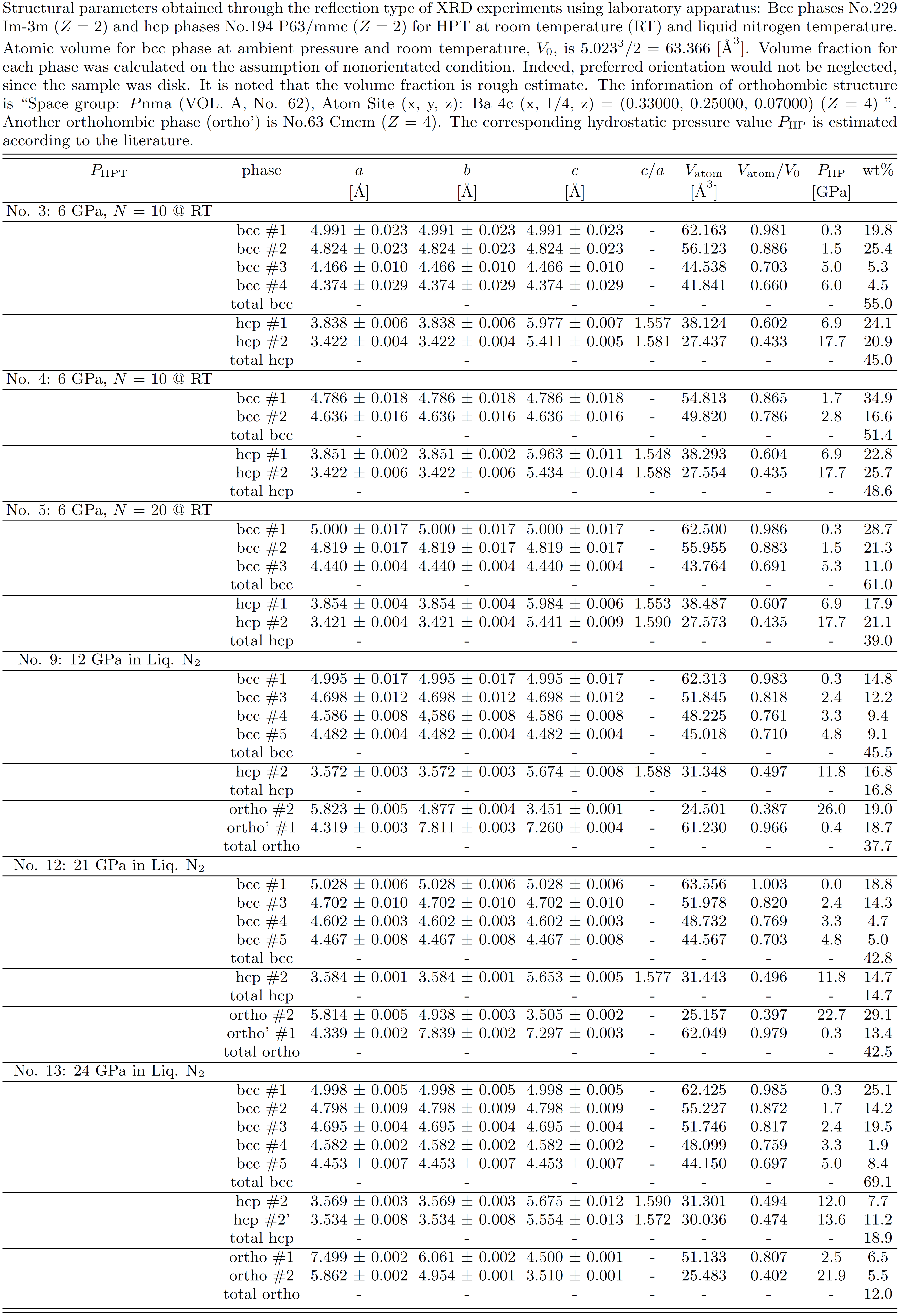


Extended Data Table 3 Summary of the thrubeam type of XRD analyses at SPring-8.
